# Epidermal growth factor receptor signalling in human breast cancer cells operates parallel to estrogen receptor α signalling and results in tamoxifen insensitive proliferation

**DOI:** 10.1186/1471-2407-14-283

**Published:** 2014-04-23

**Authors:** Marja Moerkens, Yinghui Zhang, Lynn Wester, Bob van de Water, John HN Meerman

**Affiliations:** 1 Leiden Academic Centre for Drug Research (LACDR), Department of Toxicology, Leiden University, Einsteinweg 55, 2333 CC Leiden The Netherlands

**Keywords:** Estrogen receptor, Breast cancer, Tamoxifen resistance, Epidermal growth factor receptor, PI3K/Akt signalling

## Abstract

**Background:**

Tamoxifen resistance is a major problem in the treatment of estrogen receptor (ER) α -positive breast cancer patients. Although the mechanisms behind tamoxifen resistance are still not completely understood, clinical data suggests that increased expression of receptor tyrosine kinases is involved. Here, we studied the estrogen and anti-estrogen sensitivity of human breast cancer MCF7 cells that have a moderate, retroviral-mediated, ectopic expression of epidermal growth factor receptor (MCF7-EGFR).

**Methods:**

Proliferation of MCF7-EGFR and parental cells was induced by 17β-estradiol (E2), epidermal growth factor (EGF) or a combination of these. Inhibition of proliferation under these conditions was investigated with 4-hydroxy-tamoxifen (TAM) or fulvestrant at 10^-12^ to 10^-6^ M. Cells were lysed at different time points to determine the phosphorylation status of EGFR, MAPK_1/3_, AKT and the expression of ERα. Knockdown of target genes was established using smartpool siRNAs. Transcriptomics analysis was done 6 hr after stimulation with growth factors using Affymetrix HG-U133 PM array plates.

**Results:**

While proliferation of parental MCF7 cells could only be induced by E2, proliferation of MCF7-EGFR cells could be induced by either E2 or EGF. Treatment with TAM or fulvestrant did significantly inhibit proliferation of MCF7-EGFR cells stimulated with E2 alone. EGF treatment of E2/TAM treated cells led to a marked cell proliferation thereby overruling the anti-estrogen-mediated inhibition of cell proliferation. Under these conditions, TAM however did still inhibit ERα- mediated transcription. While siRNA-mediated knock-down of EGFR inhibited the EGF- driven proliferation under TAM/E2/EGF condition, knock down of ERα did not. The TAM resistant cell proliferation mediated by the conditional EGFR-signaling may be dependent on the PI3K/Akt pathway but not the MEK/MAPK pathway, since a MEK inhibitor (U0126), did not block the proliferation. Transcriptomic analysis under the various E2/TAM/EGF conditions revealed that E2 and EGF dependent transcription have little overlap and rather operate in a parallel fashion.

**Conclusions:**

Our data indicate that enhanced EGFR-driven signalling is sufficient to overrule the TAM- mediated inhibition of E2-driven cell proliferation. This may have profound implications for the anti-estrogen treatment of ER-positive breast cancers that have increased levels of EGFR.

## Background

Breast cancer is the most common cancer among women worldwide. Despite the improvement in treatment, therapy resistance remains a major problem in the clinic. Endocrine therapy has become the most important treatment option for women with estrogen receptor (ER) α -positive breast cancer, which is approximately 70% of all breast tumours. The ERα - antagonist tamoxifen is commonly used with these ERα-positive breast cancers. Unfortunately, around 40% of all ERα-positive patients do not respond to tamoxifen treatment (*de novo* resistance) [1]. Furthermore, most patients that initially respond to tamoxifen treatment eventually develop resistance (acquired resistance) [2,3].

Clinical data indicate that tamoxifen resistant breast cancers often have an increased expression of the receptor tyrosine kinase (RTK) epidermal growth factor (EGF) receptor (EGFR/ERBB1) and its family member ERBB2 [1,4,5]. Also increased activation of their downstream target mitogen activated protein kinase (MAPK) leading to increased phosphorylation of the estrogen receptor on serine 118 or serine 167, have been found [6-8]. Because MAPK can be activated downstream from EGFR and/or ERBB2 and may phosphorylate the ERα at serine 118, together these observations suggest that the EGFR/ERBB2 signalling pathways might play a role in tamoxifen resistance.

The above clinical findings are confirmed by several *in vitro* studies which show that continuous culturing of the human breast cancer cell line MCF7 in the presence of the anti- estrogen tamoxifen or fulvestrant increases EGFR and ERBB2 expression and the activation of downstream signalling kinases (e.g. MAPK) [9-11]. This is in contrast to another study in which no change in the EGFR/ERBB2 signalling pathway upon long term tamoxifen treatment is observed [12]. Nevertheless, in the latter study an increased MAPK phosphorylation upon tamoxifen stimulation and an enhanced ERα-EGFR interaction were observed [12]. In all studies the antagonistic effect of tamoxifen could be restored by co- treatment with tyrosine kinase inhibitors against either the EGFR or against MAPK and PI3K/Akt [9-13]. Even more evidence for a role of EGFR and ERBB2 in tamoxifen resistance comes from *in vivo* experiments in mice. Masserweh et al. showed that EGFR and ERBB2 expression was markedly increased when MCF-7 xenograft tumours became tamoxifen resistant compared to control estrogen-treated tumours [14]. Together these observations suggest that the EGFR/ERBB2 signalling pathways might play a role in tamoxifen resistance.

Several *in vitro* studies show down regulation of ERα due to signalling by highly over expressed EGFR/ERBB2 pathway components [1,15-17], resulting in *de novo* or acquired tamoxifen resistance. Also in clinical studies, an inverse correlation between EGFR and ERα expression in tamoxifen resistant patients has been reported [5,6,18-20]. However, expression of both ERα and EGFR was observed in at least 50% of the patients [20]. Furthermore, in a meta analysis involving >5000 patients, EGFR positivity was observed in 4-51% (mean 29%) of ERα-positive tumors and in 29-91% (mean 59%) of ERα-negative tumors [21]. No correlations with tamoxifen were reported. In addition, several *in vitro* studies showed no down regulation of the ERα in cell lines that were long-term cultured in the presence of tamoxifen [9,10,22]. Thus, it appears that high expression of EGFR may down regulate ERα, while more moderate levels of EGFR are found in ERα-positive tumors. In this paper we focus on the latter situation and have investigated the mechanisms responsible for anti-estrogen resistance in this situation.

Despite all research done, the mechanism by which over expression of receptor tyrosine kinases induce anti-estrogen resistance is still unclear. For instance, some studies suggest that increased EGFR signalling itself induces anti-estrogen resistance [23-26], while in contrast others suggest that increased crosstalk between ERα and RTKs might be responsible [12,14,22,27-30]. Furthermore, other data also suggest a role for ERα phosphorylation by RTK downstream signalling, in anti-estrogen resistance [9,31-34]. The diversity of the explanations for the effect of RTKs on tamoxifen resistance may suggest a very complex mechanism behind the anti-estrogen resistance. Typically, these above mentioned studies are performed in anti-estrogen resistant breast tumour cell models that are created by long term culturing of human breast cancer cells in the presence of different anti- estrogens. This allows adaptation of the cells to reduced pro-mitogenic signals and may result in selection of cells with increased levels and/or activation of EGFR/ERBB2 [9,10,22,26]. However, other cellular programs may have changed in these anti-estrogen resistant cells as well which also may contribute to acquired tamoxifen resistance. Therefore, studies using isolated EGFR expression are required.

In this study we created human breast cancer MCF7 cells that ectopically express human EGFR (MCF7-EGFR) with a 3-fold induction compared to wild type MCF7 cells, allowing the study of EGFR exclusively in the context of anti-estrogen activity of tamoxifen. Importantly, in these cells EGFR activity is low under basal conditions, but is greatly enhanced by EGF treatment. This enhanced signalling leads to loss of anti-proliferative effect of tamoxifen. In contrast, classic genomic ERα signalling remains anti-estrogen sensitive. Genome-wide transcriptomic analysis showed the existence of specific E2 and EGF induced transcriptional programs that do not significantly overlap and operate in a parallel fashion.

Our data suggest that ER-positive breast cancer with a moderate EGFR expression would also be intrinsic resistant to anti-estrogens. First line combined therapy of ER/EGFR positive breast cancer with EGFR inhibitors and tamoxifen would therefore be more effective.

## Methods

### Materials

Antibodies against ERα (sc-543), and EGFR (sc-03) were from Santa Cruz Biotechnology (Heidelberg, Germany); antibodies against phosphorylated Akt (9271S), mitogen activated protein kinase 42–44 (MAPK) and phosphorylated MAPK (9101 and 137 F5), and phosphorylated EGFR (4407) were from Cell Signalling Technologies (Leiden, The Netherlands); antibody for Akt was a kind gift from P. Coffer (UMC, Utrecht, The Netherlands). For analyzing phosphorylated proteins the Western-Star immunodetection kit (Tropix kit) from Applied Biossytems (Foster City, CA, USA) was used. TAM, fulvestrant, E2, EGF, and the protein dye sulforhodamin B (SRB) were from Sigma Aldrich (St Louis, MO, USA). Mitogen-activated kinase kinase (MEK) inhibitor U0126 (V-112A) was from Promega (Leiden, The Netherlands); Phosphoinositide 3-kinase (PI3K) inhibitor BEZ235 (S1009) was from Selleck (Houston, TX, USA).

### Cell culture

All cells were cultured in RPMI 1640 medium (Gibco, Life Technologies, Grand Island, NY, USA) supplemented with 10% fetal bovine serum (FBS) and penicillin/streptomycin (25 Units/mL each) at 37°C and 5% carbon dioxide. For estrogen deprivation, cells were cultured for 48 hrs in starvation medium consisting of phenol red free RPMI 1640 medium (Gibco) supplemented with 5% charcoal dextran treated fetal bovine serum (CDFBS) (HyClone, Thermo Scientific, Waltham, MA, USA) and penicillin/streptomycin.

### Establishment of MCF EGFR cells

Retroviral transduction of MCF7 cells with a pMSCV-blast-hEGFR retroviral vector, kindly provided by Dr. E. Danen (Leiden Academic Centre for Drug Research, The Netherlands) [35], followed by blasticidin selection (12.5 μg/ml) was used to generate MCF7-hEGFR cells. After 7 passages of continuous selection with blasticidin, EGFR transduced cells were harvested by fluorescence-activated cell sorting (FACS). Cells were maintained at 10 μg/ml blasticidin.

### Proliferation assay

Parental MCF7 and MCF7-EGFR cells were plated in 96- wells plates (Costar, Fisher Scientific, Waltham, MA, USA) at a density of 10.000 cells/well and allowed to attach overnight and maintained in starvation medium for 48 hrs. Subsequently, growth factors were added (E2, EGF, TAM, etc.) and cells were allowed to proliferate for 5 days. The cells were fixed and stained using the colorimetric sulforhodamin B (SRB) assay [36]. In short, cells were fixed with trichloroacetic acid at 4°C for 1 hour, washed five times with tap water and air-dried. Next, the cells were stained with SRB in 1% acetic acid at room temperature for 30 min. Plates were washed five times with 1% acetic acid and air-dried overnight. Bound SRB was solubilised with 100 μL 10 mM aqueous unbuffered Tris solution (pH > 10) and absorbance was measured at 540 nm. All data represent the average ± SEM of three independent experiments each performed with triplicate wells.

In a control experiment (Additional file 1: Figure S1), cell proliferation was determined by staining cellular DNA in 96-well tissue cultures plates with bisbenzimidazole (Hoechst 33258) as described [37]. Briefly, the plates were emptied of media and stored frozen. Subsequently 100 μL distilled water was added to each well and frozen again. Thereafter, they were stained with Hoechst 33258 in 5 mM Tris, 0.5 mM EDTA, 1 M NaCl pH 7.4. The assay yielded a linear standard curve for DNA fluorescence versus cell number in a range appropriate for our experiment.

### Immunoblotting

Estrogen depleted parental MCF7 and MCF7-EGFR cells plated in 60-mm dishes were treated with different stimuli after a 2 hr serum starvation period. After stimulation, cells were placed on ice and washed twice with ice-cold PBS and once with ice cold TSE (10 mM Tris, 250 mM Sucrose, and 1 mM EGTA). Next, cells were lysed in 60 μL TSE plus inhibitors (1 mM DTT, 10 μg/mL leupeptin, 10 μg/mL aprotinin, 1 mM vanadate, 50 mM sodium fluoride, 1 mM PMSF) and lysates were placed in cold 1 mL eppendorf tubes. After pulse sonication samples were stored at -20°C until electrophoresis. Proteins were separated by electrophoresis (7.5% acrylamide gel) followed by transfer to PVDF membrane (Millipore, Billerica, MA, USA). After blocking with 5% bovine serum albumin (BSA) (Invitrogen, Grand Island, NY, USA) and primary and secondary antibody staining, protein bands were visualized by scanning the membrane on a Typhoon 9400 (GE Healthcare, Fairfield, CT, USA).

### Immunofluorescent microscopy

Parental MCF7 and MCF7-EGFR cells plated on glass coverslips were fixed with 4% formaldehyde for 10 min at room temperature, washed three times with PBS and then blocked with TBP (10% Triton, 1% BSA in PBS pH 7.4) for 1 hour at room temperature. Primary antibodies diluted in TBP were added for incubation overnight at 4°C. Thereafter, secondary antibody conjugated with Alexa488 was added together with Hoechst33258 (2 μg/ml) for 30 min at room temperature in the dark and post-fixated with 4% formaldehyde for 5 min. After washing with TBP and PBS, coverslips were mounted on a glass slide using Aqua- Poly/Mount (Polysciences Inc., Warrington, PA, USA).

### Small interfering RNA (siRNA)-based knockdown

Knockdown of target genes was established by a reverse transfection using smartpool siRNAs according to the manufacture’s protocol (Dharmacon, Pittsburgh, PA, USA) using Dharmafect 4 reagent and with final siRNA concentration of 50 nM.

### Luciferase reporter assays

Parental MCF7 and MCF7-EGFR cells were plated at a density of 40.000 cells/well in a 48- wells plate in culture medium without antibiotics. The next day cells were transfected with 0.16 μg ERE-tk-luciferase plasmid (kind gift of R. Michalides, Netherlands Cancer Institute, Amsterdam) using Lipofectamine Plus reagents (Invitrogen) according to manufacturer’s protocol. After 3 hours incubation medium was replaced with starvation medium. Cells were cultured for 48 hrs before treatment with different compounds. The medium was discarded after 12 hrs and cells were washed once with PBS and then lysed with 1x passive lysis buffer, from the Dual-Luciferase kit (Promega, Madison, WI, USA). Luciferase activity was measured using the Dual- Luciferase kit (Promega, Madison, WI, USA) on a luminometer (CentroXS3 LB960, Berthold Technologies, Bad Wildbad Germany).

### Transcriptomics analysis

For microarray analysis of gene expression, MCF7-EGFR cells were seeded at 60% confluence in 6-cm plates and subjected to three-day starvation in 5% charcoal/dextran- stripped fetal bovine serum medium prior to treatments with TAM (10 μM), E2 (10 nM) and EGF (100 ng/mL) in triplicate. After 6 hours, total RNA was extracted using a RNA isolation kit (Ambion, Inc., Austin, TX, USA). Affymetrix 3′ IVT Express Kit (Affymetrix, Santa Clara, CA, USA) was used to synthesize biotin-labeled cRNA, and this was hybridized to a Affymetrix HG-U133 PM Array plate. Raw expression data were obtained by probe summarization and background correction according to the robust multiarray averaging method [38]. Median normalization of raw expression data and identification of differentially expressed genes using a random variance t-test was performed using BRB-ArrayTools [39] version 4.1.0 Beta 2 Release (developed by Dr. Richard Simon and BRBArrayTools Development Team members). Corrections for multiple testing were made by calculating the false discovery rates according to Benjamini & Hochberg [40]. Affymetrix probesets were annotated with Netaffx Annotation build 30 (dated 08-20-2010).

### Statistical analysis

Student’s t-test was used to determine if there was a significant difference between two conditions/treatments (p < 0.05). Significant differences are indicated in the figures.

## Results

### EGFR over expression in MCF7 cells enhances downstream MAPK and Akt signalling

**Figure 1:**
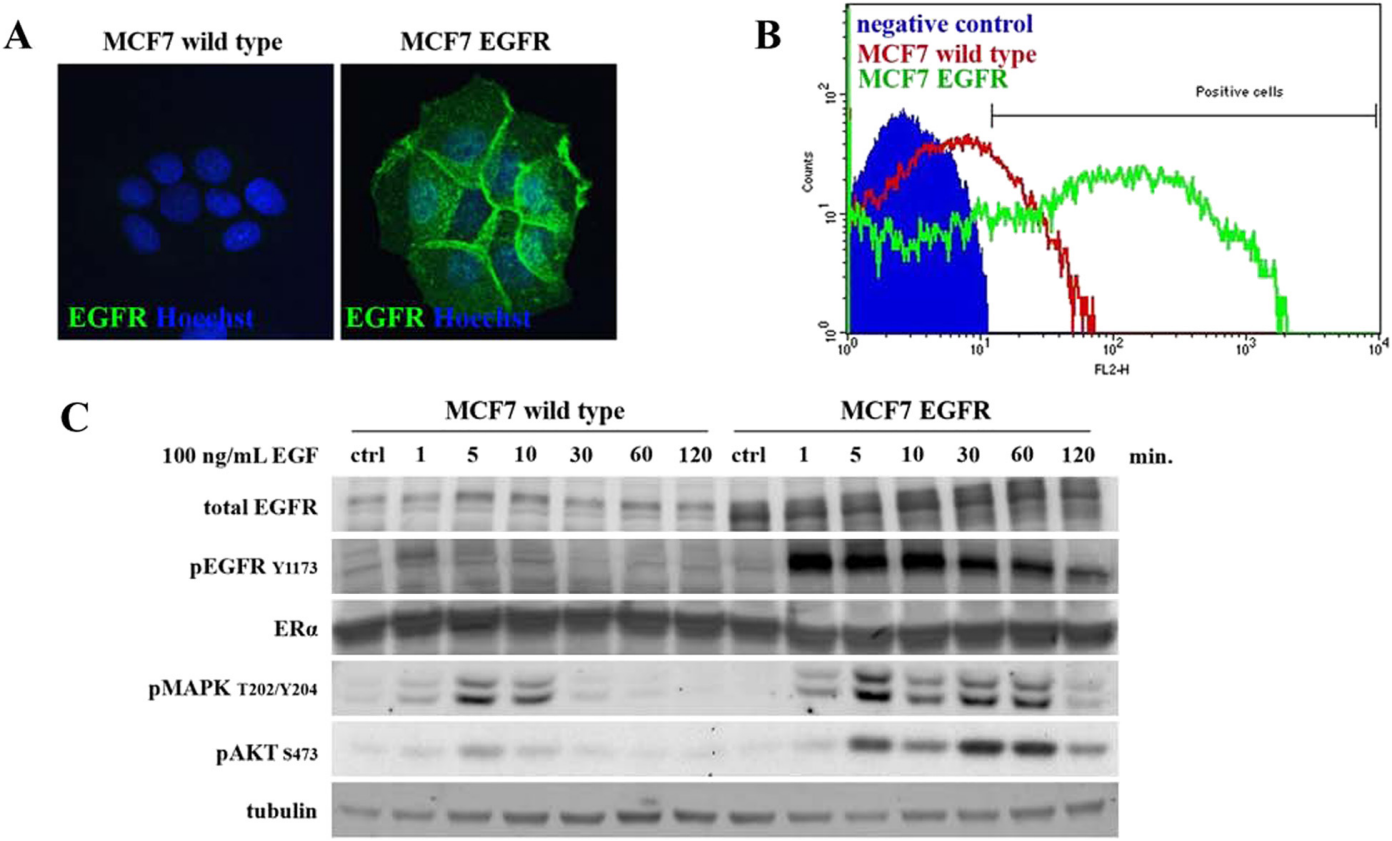
**Retroviral-induced EGFR over expression in MCF7 human breast cancer cells enhances downstream signalling*.*** EGFR expression was determined in parental MCF7 and MCF7-EGFR cells by immunofluorescence **(A)** and FACS analysis **(B)**. To determine downstream EGFR signalling, starved MCF7 parental and MCF7-EGFR cells were stimulated with EGF (100 ng/mL). Cell lysates were collected and analyzed by western blot for the phosphorylation status of EGFR, MAPK_1/3_ and Akt as well as the expression of ERα **(C)**.

To investigate the role of EGFR on anti-estrogen resistance, we established ectopic human EGFR expression in human MCF7 breast cancer cells. Immunofluorescent staining of these MCF7-EGFR cells showed an intense plasma-membrane EGFR staining (Figure 1A) in contrast to the parental MCF7 cells. Furthermore, FACS analysis also demonstrated a clear increase of EGFR expression in the established MCF7-EGFR cell line (Figure 1B). Next, we determined the functionality of ectopically expressed EGFR by analyzing the downstream signalling upon EGF stimulation. Cells were serum starved for 2 hours prior to EGF stimulation (100 ng/mL). The MCF7-EGFR cells showed a long lasting (>120 min) increased phosphorylation of the EGFR upon EGF stimulation (Figure 1C). This EGFR activation was associated with enhanced activation of the downstream kinases MAPK_1/3_ and Akt (Figure 1C). Importantly, no difference in ERα protein expression between the two cell lines was observed at 2 hr (Figure 1C), 2 days and 5 days after continuous EGF stimulation (Additional file 2: Figure S2), indicating that this level of EGFR expression does not affect ERα levels.

### MCF7-EGFR proliferation can be induced by both estrogen and EGF

**Figure 2:**
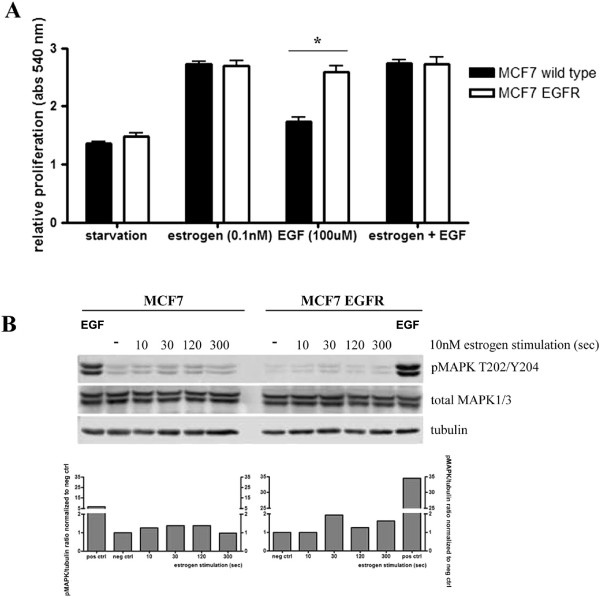
**EGFR over expression does not influence estrogen-dependent proliferation*.*** To investigate the proliferation induced by either estrogen or EGF both, parental MCF7 and MCF7-EGFR cells, were cultured in phenol red free medium with 5% charcoal treated serum for 48 hours, followed by an exposure to 0.1 nM E2, 100 ng/mL EGF or a combined exposure. The control cells were exposed to DMSO only. Cells were left to proliferate for 5 days and then fixed with 50% trichloroacid (TCA). Fixed cells were stained with sulforhodamin B, which absorption was measured at 540 nm **(A)**. Graphs represents the average relative proliferation ± SEM of three independent experiments, * indicates significant difference of p < 0.05. To determine the role for the fast non-genomic effects of ERα, starved MCF7 parental and MCF7-EGFR cells were exposed to 10 nM E2 for the indicated times before lysates were collected and analyzed by western blot for the phosphorylation status of MAPK_1/3_**(B)**. – and + indicate negative control (DMSO) and positive control (EGF, 100 ng/mL).

Both MCF7 parental and MCF7-EGFR cells showed a clear estrogen-dependent increase in proliferation (Figure 2A). However, stimulation with EGF induced proliferation of only the MCF7-EGFR cells, which was almost the same as E2-induced proliferation (Figure 2A). Furthermore, the E2- induced proliferation did not increase by additional EGF stimulation (Figure 2A), indicating lack of synergy between EGF and E2 at the concentrations used.

We also investigated non-genomic effects of ERα signalling by analyzing phosphorylation of MAPK_1/3_ after E2 stimulation (10 nM) in estrogen (48 hrs) and serum (2 hrs) starved cells. The parental MCF7 and MCF7-EGFR cells showed a small increase (1.5 and 2 fold respectively) in MAPK_1/3_ activation 30 seconds after E2 stimulation (Figure 2B). However, this was much smaller than the 5 and 35 fold increase by EGF stimulation. Even when the estrogen stimulation was prolonged, MAPK_1/3_ activation did not further increase (data not shown). These results may suggest that non-genomic effects of ERα in relation to MAPK signalling might not be very important in MCF7-EGFR cells.

### Ectopic EGFR expression provides resistance to the anti-estrogen tamoxifen

**Figure 3:**
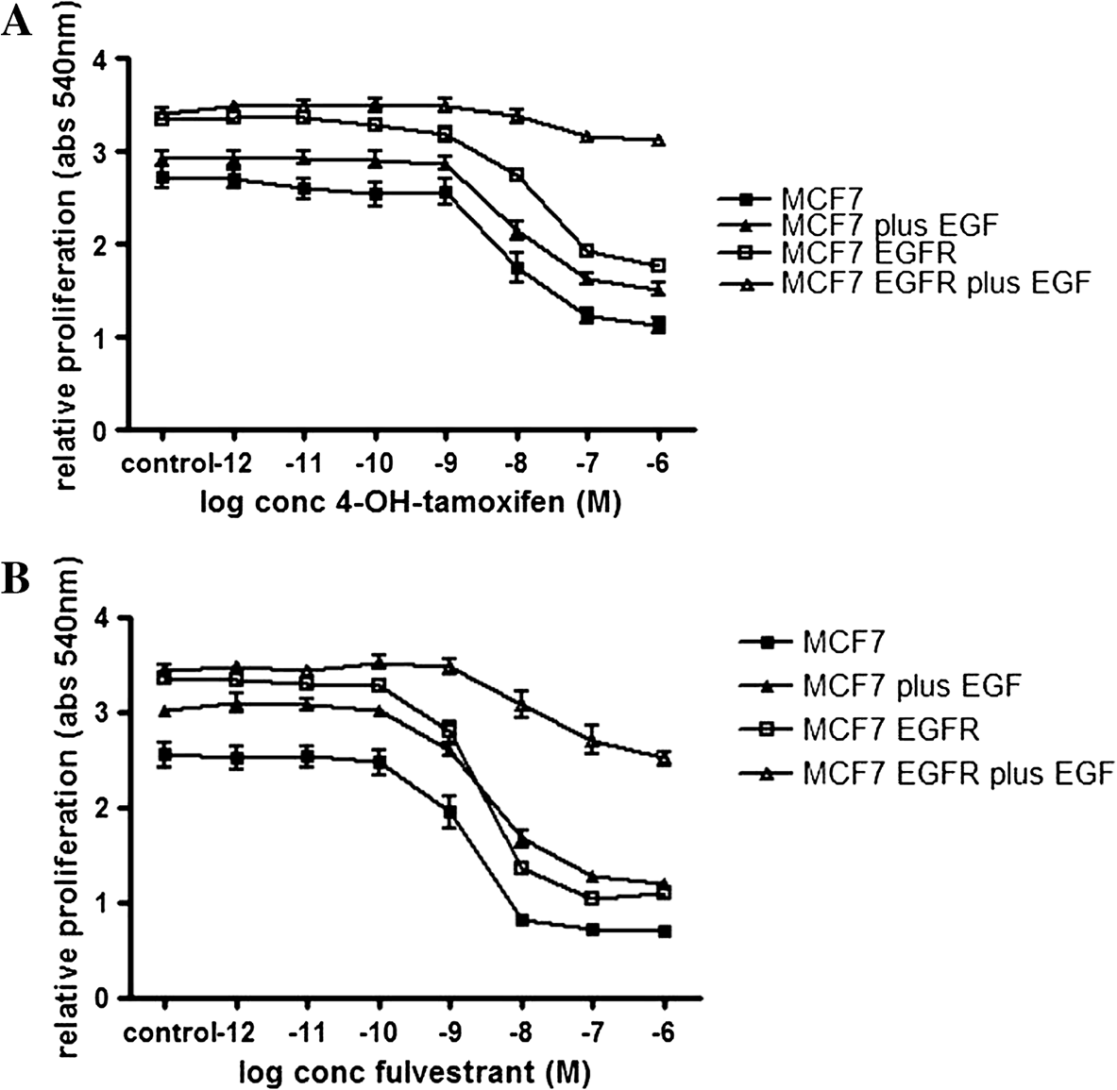
**EGFR over expression induces tamoxifen and fulvestrant resistance.** Parental MCF7 and MCF7-EGFR cells were estrogen starved 48 hours prior to a 5 day proliferation period in the presence of 0.1 nM E2 with a concentration series TAM **(A)** or fulvestrant **(B)**, with or without 100 ng/mL EGF. Afterwards, cells were fixed with 50% TCA and stained with sulforhodamin B, which absorbance was measured at 540 nm. Graphs represent the average ± SEM of three independent experiments.

Next, we determined the effect of EGFR over expression on the sensitivity towards the anti-estrogen tamoxifen. Cells were estrogen-depleted for 48 hrs and then exposed to a concentration series of TAM plus a fixed concentration E2 (0.1 nM) with or without EGF (100 ng/mL). After 5 days, proliferation was determined. As expected, TAM treatment resulted in a dose-dependent inhibition of proliferation of parental MCF7 cells (Figure 3A). The MCF7-EGFR cells without EGF showed a similar dose-dependent inhibition of proliferation upon TAM treatment. However, when the EGFR is activated by EGF exposure, the MCF7-EGFR cells were no longer sensitive to TAM. As the SRB assay that we used for determining cell proliferation is based on measuring total cell proteins, any change in cellular protein content by EGF exposure may have influenced our results. Therefore, we performed an independent experiment where we determined cell proliferation by measuring total cellular DNA (see Methods). The results are in agreement with the SRB assay and confirm that MCF7-EGFR cells after EGF exposure are no longer sensitive to TAM (Additional file 1: Figure S1).

**Figure 4:**
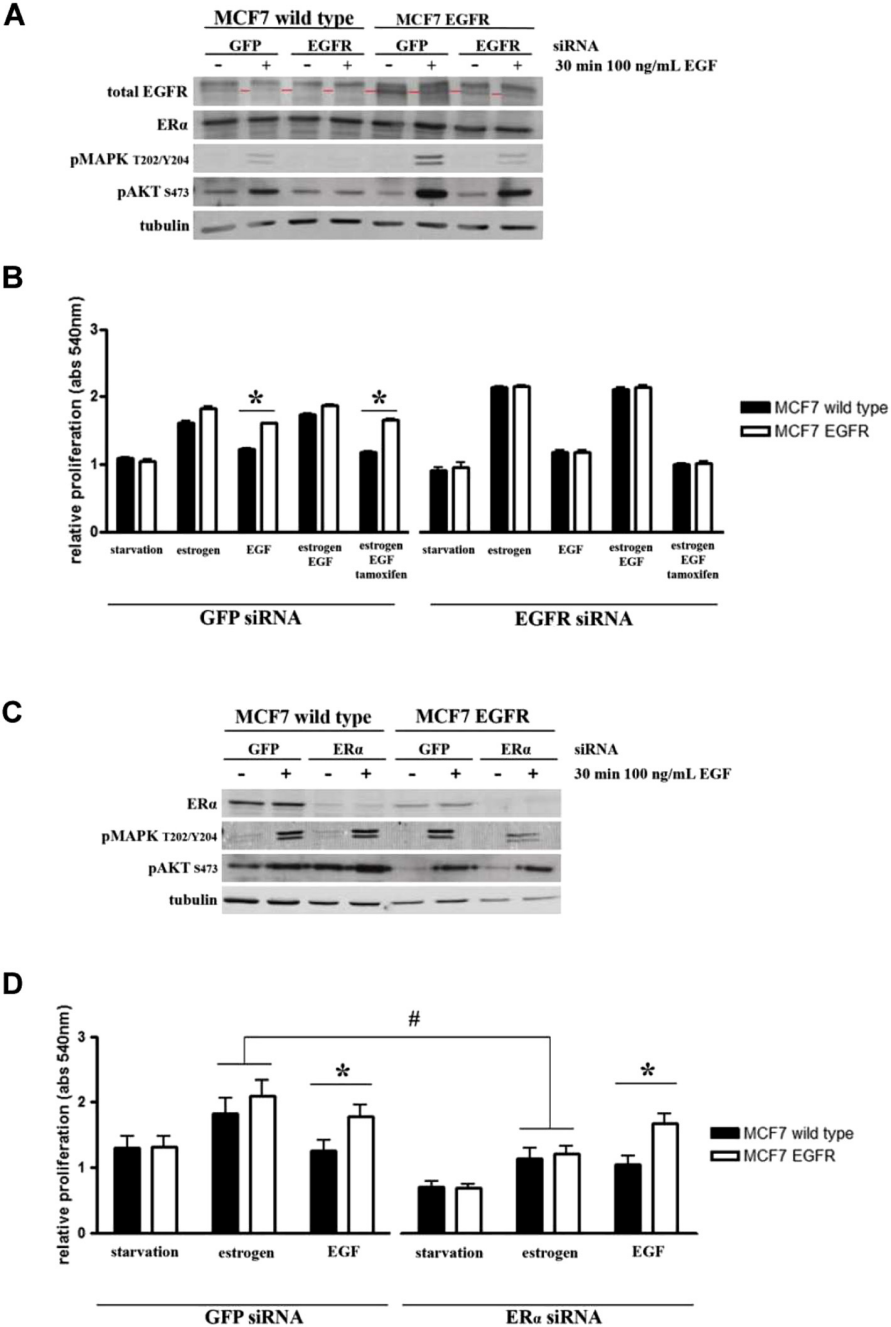
**Knock down of EGFR reverses tamoxifen resistance of MCF7-EGFR cells and is ERα independent.** EGFR **(A)** and ERα **(C)** knockdown in MCF7 parental and MCF7-EGFR cells was established using siRNA. Knock down efficiency and the effect of EGFR knock down on phosphorylation status of MAPK1/3 and Akt after 30 min EGF (100 ng/mL) exposure was analysed on western blot. GFP siRNA was used as control. **(A, C)** After 48 hours starvation knock down cells were exposed to 0.1 nM E2 plus 100 nM TAM and 100 ng/mL EGF. Proliferation was measured after 5 days using sulforhodamin B absorbance at 540 nm **(B, D)**. Graphs represent the average ± SEM of three **(A, B)** or four **(C, D)** individual experiments, * indicates significantly different at p < 0.05; # indicates significantly different at p < 0.01.

Subsequently, we tested whether the EGF-mediated protection against TAM was dependent on the EGFR signalling. For this purpose we performed siRNA-based knockdown of EGFR in both the MCF7 and MCF7-EGFR cells. After a starvation period of 48 hrs, cells were stimulated with either E2 (0.1 nM), EGF (100 ng/mL), E2 and EGF, or E2 plus EGF and TAM (100 nM). Western blot analysis showed a 60% knock down of EGFR compared to control GFP siRNA, which led to decreased activation of the downstream kinases MAPK_1/3_ and Akt upon EGF stimulation in both MCF7 parental and MCF7-EGFR cells (Figure 4A). Furthermore, as expected, EGF-induced proliferation of MCF7-EGFR cells decreased significantly in cells with a knock down of EGFR compared to cells with a control siRNA (Figure 4B). Knock down of EGFR in the MCF7-EGFR cells resulted in almost complete re- sensitization towards TAM treatment (Figure 4B). This indicates that the EGFR signalling pathway is dominant over the TAM-induced inhibition of estrogen-driven proliferation.

### MCF- EGFR cells show resistance to the anti-estrogen fulvestrant

Next, we determined the sensitivity of the MCF7-EGFR cells towards another clinically relevant anti-estrogen, namely fulvestrant. In contrast to tamoxifen, fulvestrant binds, blocks and *degrades* the ERα [41]. Therefore, all ERα-dependent pathways are expected to be inhibited by fulvestrant. Cells were estrogen-depleted for 48 hrs and then exposed to a concentration series of fulvestrant plus a fixed concentration E2 (0.1 nM) with or without EGF (100 ng/mL). The MCF7 parental cells showed an almost complete, dose-dependent inhibition of proliferation by fulvestrant that was independent of EGF treatment (Figure 3B). This is similar to the effect of TAM.

Treatment of the MCF7-EGFR cells with fulvestrant resulted in a dose-dependent inhibition of proliferation as well (Figure 3B). However, co-treatment of these cells with EGF decreased the inhibitory effect of fulvestrant, similar to the effect on TAM.

### Knock down of ERα blocks E2- but not EGF-induced proliferation

To determine whether EGF-induced EGFR signalling resulting in tamoxifen resistance involves ERα or not, we introduced a siRNA targeting ERα in both parental MCF7 and MCF7-EGFR cells, which resulted in 70% ERα knock down (Figure 4C). This ERα knockdown did not decrease the activation of MAPK_1/3_ or Akt upon EGF stimulation (Figure 4C). However, estrogen-induced proliferation was greatly reduced in ERα knockdown cells compared to control GFP siRNA (Figure 4D), although some E2-driven proliferation was still observed, possibly related to residual ERα protein levels due to no full ERα knockdown. EGF-induced proliferation was not significantly affected by ERα knockdown in neither MCF7 parental nor MCF7-EGFR cells. These results indicate that EGFR signalling pathway can maintain proliferation in the absence of ERα in MCF7-EGFR cells.

### MEK/MAPK pathway is not responsible for EGFR-mediated proliferation and tamoxifen “resistance” of MCF7-EGFR cells

**Figure 5:**
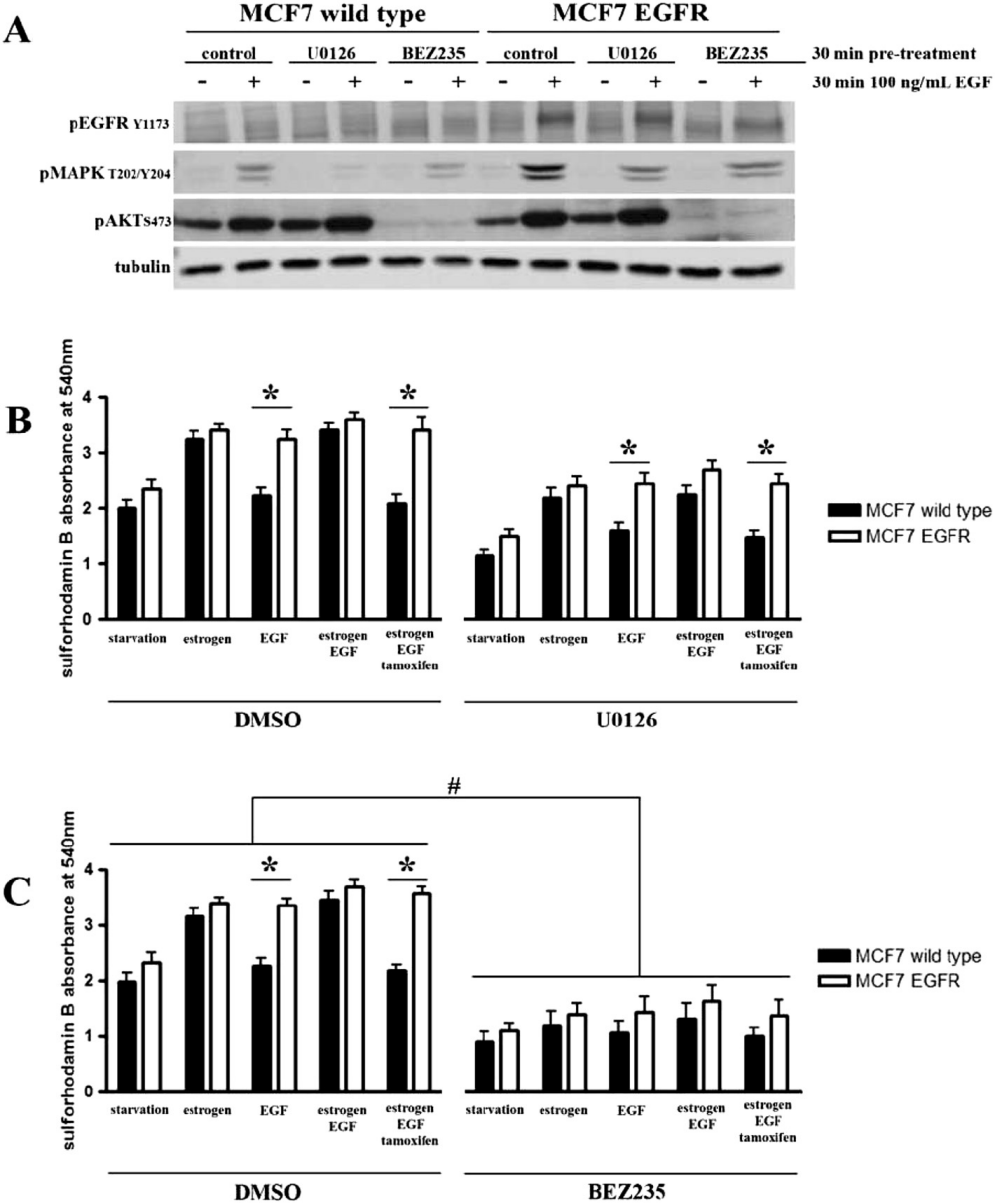
**MCF7-EGFR tamoxifen resistance involves PI3K/Akt pathway.** Parental MCF7 and MCF7-EGFR cells were starved for 48 hrs before pre-treatment with either the MEK inhibitor U0126 (10 μM) or the PI3K inhibitor BEZ235 (1 μM) for 30 min. The effect of inhibition on EGF-induced activation of MAPK_1/3_ and Akt was analyzed on western blot **(A)**. Following U0126 and BEZ235 pre-treatment cells were exposed to 0.1 nM E2, 100 nM TAM and 100 ng/mL EGF. Proliferation was measured after 5 days using sulforhodamin B absorbance at 540 nm **(B, C)**. Graphs represent the average ± SEM of three independent experiments, * indicates significant difference of p < 0.05, # indicates significant difference of p < 0.01.

To determine the downstream signalling that defines the EGFR-mediated proliferation and resistance to tamoxifen we treated our cells with an inhibitor of MEK_1/2_ (U0126, 10 μM) and an inhibitor of PI3K (BEZ235, 1 μM) and measured the proliferation of MCF7 parental and MCF7-EGFR cells treated with E2 (0.1 nM), EGF (100 ng/mL), E2 and EGF, or E2 plus EGF and TAM (100 nM). Western blot analysis showed reduced MAPK_1/3_ activation upon U0126 treatment and reduced Akt activation upon BEZ235 treatment in both parental MCF7 and MCF7-EGFR cells (Figure 5A). Treatment with the MEK_1/2_ inhibitor resulted in decreased proliferation of serum starved MCF7 parental as well as MCF7-EGFR cells compared to control (Figure 5B). Similarly, proliferation after E2, EGF, E2 + EGF, and E2 + EGF + TAM stimulation was decreased as well compared to control (Figure 5B). The decrease in proliferation, however, was comparable to the decrease in proliferation in the starvation conditions. The MEK_1/2_ inhibitor did not change the effect of TAM on proliferation of parental MCF7 and MCF7-EGFR cells in the presence of E2 and EGF (Figure 5B). These results suggest that the MEK/MAPK pathway is not responsible for the apparent tamoxifen resistance in MCF7-EGFR cells. Treatment with the PI3K inhibitor BEZ235 almost completely blocked proliferation induced by E2, EGF, or by a combination of the two (Figure 5C) in parental MCF7 and MCF7-EGFR cells. BEZ235 also has an effect on starved control cells, which is likely related to remaining background PI3K signalling activity mediated by cell adhesion signalling and/or autocrine responses. Yet, altogether our data indicate that tamoxifen resistant cell proliferation mediated by the conditional EGFR- signalling may be dependent on the PI3K/Akt pathway but not the MEK/MAPK pathway, since strong Akt activation is observed after EGF stimulation of MCF7-EGFR cells (Figure 1C) and a MEK inhibitor (U0126), did not block the proliferation.

### Overexpression of EGFR does not overcome tamoxifen inhibition on transcriptional level

Tamoxifen resistance may be related to altered regulation of ERα-mediated transcriptional activity [14,22]. Therefore, we investigated the effect of ectopic EGFR expression and tamoxifen on ERα transcription. Parental MCF7 and MCF7-EGFR cells were transiently transfected with an ERE-tk-luciferase construct. Estrogen induced ERE-luciferase activity in both parental MCF7 and MCF7-EGFR cells 4-fold which could be inhibited by tamoxifen (Additional file 3: Figure S3). Importantly, TAM inhibited E2 induced ERE-luciferase activity also after EGF stimulation in both parental MCF7 and MCF7-EGFR cells. Thus, over expression of EGFR does not block the inhibitory effect of tamoxifen on ERα transcription activation by E2_,_ as opposed to the effect on proliferation. Furthermore, EGF stimulation itself did not induce ERE-luciferase expression in MCF7 parental nor MCF7-EGFR cells (Additional file 3: Figure S3 A and B) indicating no important cross-talk between ERα and EGFR signalling pathways at the transcriptional level. Ensuing microarray gene expression analysis supported these reporter assay results (see below). In addition, we also measured ERE-luciferase expression at various times (2–12 hrs) after stimulation of parental MCF7 and MCF7 EGFR cells by EGF, with and without TAM, and these experiments also showed only little effect of EGFR signalling on transcription compared to E2, and no reinforcement of TAM on EGFR signalling (Additional file 4: Figure S4).

### Overexpression of EGFR does not induce agonistic effects of tamoxifen

It has been suggested that ERα phosphorylation by RTK downstream signalling, may alter it in such a way that tamoxifen functions as an agonst [9,33,42,43]. We therefore investigated whether enhanced EGFR signalling in our MCF7-EGFR cells led to agonistic effects of tamoxifen on MCF7 and MCF7-EGFR cell proliferation and transcription. We observed no agonistic effects of TAM after EGF stimulation on cell proliferation (Additional file 5: Figure S5), or luciferase expression (Additional file 4: Figure S4).

### Microarray gene expression analysis of E2 and EGF induced genes

**Figure 6:**
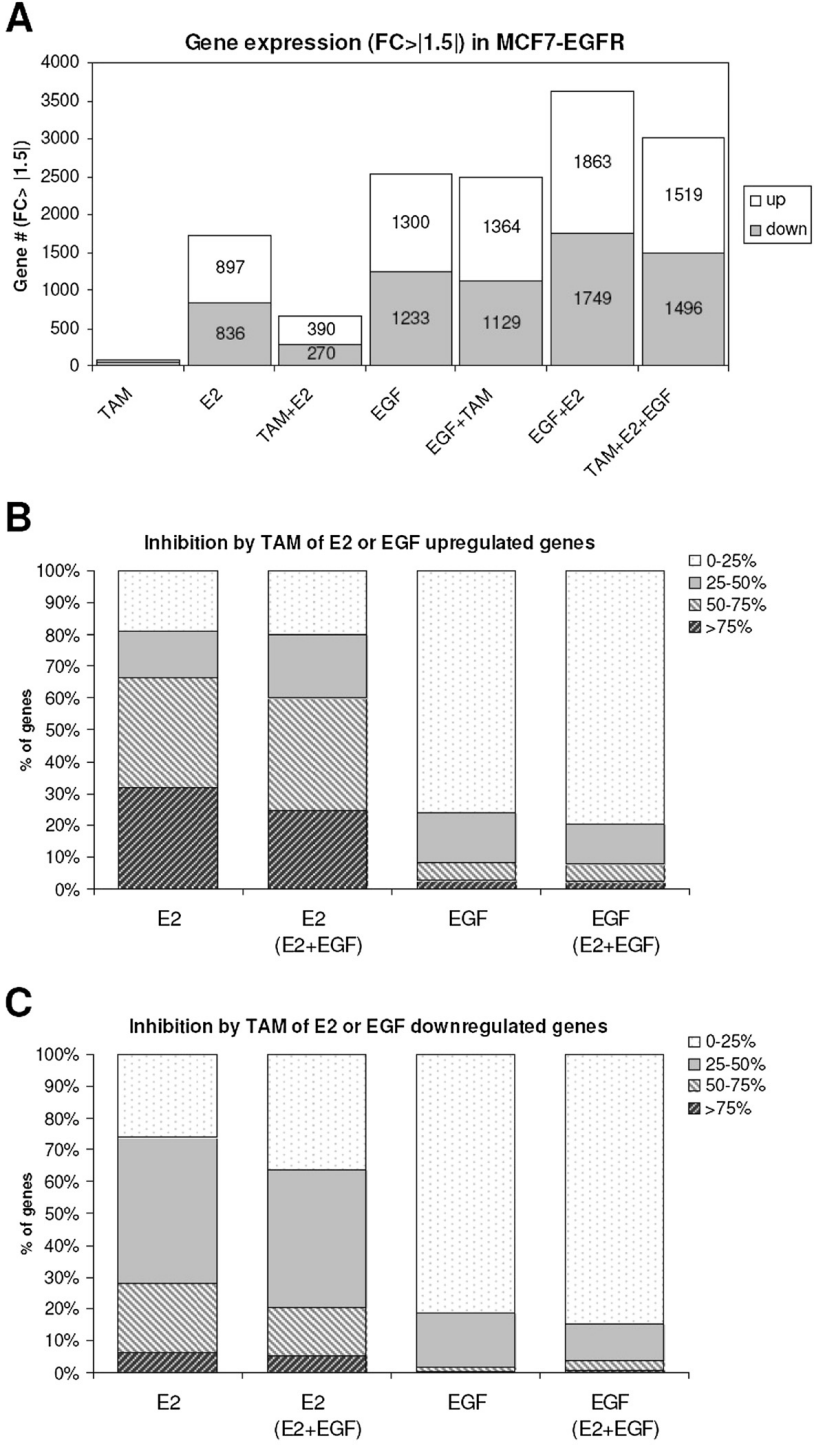
**Differentially expressed genes by E2 and EGF compared to controls and effect of TAM. (A)**. Number of genes significantly up- or down-regulated (>1.5×) in MCF7-EGFR cells 6 hr after treatment with E2 (10 nM), EGF (100 ng/ml) or E2 + EGF, with and without 10 μM TAM, compared to controls as determined by microarray gene expression analysis. **(B)** The percentage inhibition by TAM for genes >1.5× fold up regulated by exposure to E2 or EGF. **(C)** The percentage inhibition by TAM for genes >1.5× fold dow nregulated by exposure to E2 or EGF. Also shown is the inhibition by TAM of the subsets of E2 or EGF regulated genes that are also regulated by E2 or EGF under the condition of combined exposure to E2 + EGF.

Transcription analysis was performed to investigate the degree of similarity of E2 and EGF activated signalling pathways. E2 increased the expression of 897 genes by 1.5 fold in MCF7- EGFR cells after 6 hr, while a similar number of genes was 1.5 fold lower expressed compared to controls (Figure 6A). The number of genes induced or decreased by EGF was slightly higher (1300). As expected, TAM greatly reduced the number of genes 1.5 fold up- or down-regulated by E2. TAM hardly affected the number of EGF regulated genes. TAM, however, had a significant effect on the number of genes regulated by combined E2 + EGF exposure due to down regulation of E2 responsive genes (Figure 6B).

In order to further characterize the inhibitory effect of TAM on E2 regulated genes, we calculated the percentage of inhibition by TAM for each gene. The inhibition by TAM of E2 induced genes was large: the expression of more than 65% of E2 up regulated genes was inhibited by TAM by >50% (Figure 6B). Interestingly, the effect of TAM on genes up regulated by E2 under the condition of combined E2 + EGF exposure (60% inhibition >50%) was almost as big as with exposure to E2 alone. This indicates that the inhibitory effect of TAM is only slightly affected by exposure of the cells to EGF.

In general, similar observations were made for the inhibitory effect of TAM on E2 down regulated genes as for E2 up regulated genes (Figure 6C).

**Figure 7:**
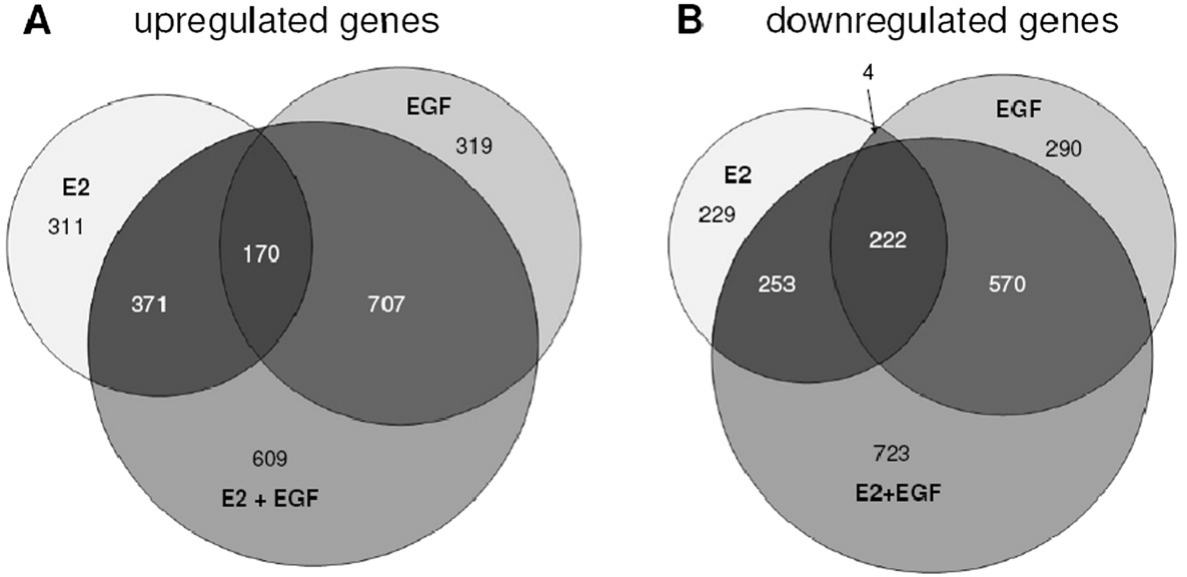
**Overlap of E2 and EGF regulated genes***.* Overlap of genes significantly up- **(A)** or down-regulated **(B)** (>1.5×) in MCF7-EGFR cells 6 hr after treatment with E2 (10 nM), EGF (100 ng/ml) or E2 + EGF as determined microarray gene expression analysis.

Further analysis of the E2 and EGF regulated genes showed that the identity of E2 and EGF induced genes are different: most genes up regulated by E2 (80%) are not induced by EGF (Figure 7). Many known E2 regulated genes such as TFF1, PGR, GREB1 and MYC belong to this class. Similarly, the majority of EGF induced genes (86%) is not induced by E2. However, there is number of genes (170) that is up regulated >1.5 fold by both E2 and EGF, and for part of these (68), there is a synergistic effect of E2 and EGF (Additional file 6: Table S1). Analysis with Metacore software (Genego, St. Joseph, MI, USA) suggests that the most important transcription factors for these genes are AR, c-JUN, c-MYC, EGR1, ESR1, HIF1A, p53 and SP1 (Additional file 6: Table S1), which is consistent with the cell proliferation pathways activated by E2 and EGF (see below).

Furthermore, there is a relatively large number of genes (609) induced by combined E2 + EGF exposure that is not induced by E2 or EGF alone. This most likely is also due to a synergistic effect of E2 and EGF because 60% of these genes are already induced by E2 or EGF alone but just below the threshold of 1.5 fold (between 1.2 and 1.5 fold).

Conversely, there is also an antagonistic effect because some of the E2 up regulated genes are down regulated by EGF, and *visa versa* (Additional file 7: Table S2). In conclusion, the majority of genes are uniquely induced by either E2 or EGF and only for a limited number of genes there is an agonistic or antagonistic effect. Similar conclusions can be drawn for E2 and EGF down regulated genes.

### E2 and EGF induced cell signalling responsible for cell proliferation

E2 and EGF induced expression of genes known to be involved in the control of cell proliferation, and these were different for E2 and EGF induced genes (Additional file 8: Table S3). Thus, an important part of the E2 induced signalling centres around activation of RB1-E2F pathway that regulates the progression through the G1 phase of the mammalian cell cycle [44]. This involves phosphorylation of RB1 by the CyclinD/CdK4/6 complex. Factors activating the CyclinD/CdK4/6 complex include CDC25A and MYC, and inhibitors include CDKN1A (p21), SMAD3, TGFB members, and CDKN2B (p15/INK4). The up- and down- regulation of these factors by E2 and/or EGFR are presented in Additional file 8: Table S3. These data clearly show that there is a general up regulation of activating factors, and a down regulation of inhibitors of CyclinD/CdK4/6 by E2. This results in activation of E2F mediated transcription which is exemplified by increased transcription of E2F regulated genes [45] such as CCNA1, CCND1, CCNE2, TK1, PCNA, DHFR, EZH2, and CDC6 (Additional file 8: Table S3). At the same time, pro-apoptosis factors (SGPL1, BIK, BMF, APAF1) are down regulated and anti-apoptotic factors (FAIM3, BCL2, IER3, HSPB) are upregulated, which contributes to cell proliferation and survival.

Interestingly, also a number of oncogenes is up regulated by E2 (MERTK, RET and its ligand ARTN), and several (putative) tumor suppressor genes are down regulated by E2 (BLNK, LATS2, RPRM) that are not, or less, regulated by EGF (Additional file 8: Table S3). Many of these E2-induced changes in gene expression could be inhibited with TAM (average inhibition >50%).

On the other hand, EGF-induced signalling relies more on activation of the RAS/RAF/MEK/MAPK/ELK1 and PI3K/Akt pathways because phosphorylation of MAPK1/3 and Akt were greatly increased after EGF stimulation of MCF7/EGFR cells (Figure 1, 5A). Consistent with this activation, transcription of FOS, EGR1 and JUNB [46-48] was increased by EGF (Additional file 8: Table S3), and also up regulation of RELB, GADD45A, ETV5, ANGPTL4, and down regulation of TOB1 and PDCD4 which is part of a MAPK signature in MCF7 cells [49] was observed. Moreover, further increase of JUN/FOS signalling may occur through cooperation with Smad3 [50] because expression of this factor is also increased several fold as is the upstream regulator of Smad signalling, TGFBR2 and its ligand TGFB2.

Because the results so far had indicated that EGFR-driven proliferation may be dependent on the PI3K/Akt pathway and to a lesser extent on the MEK/MAPK pathway, we also investigated PI3K/Akt regulated gene expression. This may be accomplished via the transcription factors, CREB and NF-κB [51,52]. Indeed, several CREB target genes [53] including oncogenes involved in RAS and JUN activation (CRKL) and inhibition of CDKNB1/p27 Kip1 and p53 activity (MLF1), an anti-apoptotic protein (MCL1), and a membrane receptor signal regulator (GEM) were increased after EGF stimulation.

Another pathway that is activated after EGF stimulation is STAT3 mediated signalling. Stat3 can be activated through EGFR signalling [54], but signalling through this pathway may also be increased because expression of both this transcription factor itself and its upstream activator, IL20 are increased after EGF stimulation (Additional file 8: Table S3). In addition, the receptor components IL6R, OSMR, GP130 and the ligand LIF are also increased which may lead to STAT3 activation through JAK2 [55].

Also after EGF stimulation (similar to E2 stimulation), there is a down regulation of pro-apoptosis factors (SGPL1, BIK, BMF, APAF1) and a tumor suppressor (BLNK), and up regulation of anti-apoptotic factors (FAIM3, HSPB), which may contribute to cell proliferation and survival.

## Discussion

Resistance to endocrine therapy in breast cancer remains a major problem in the clinic. The mechanism behind this resistance is complex and it is still unclear whether tamoxifen resistance is based on 1) decreased transcription inhibition and consequent proliferation inhibition, 2) decreased proliferation inhibition via non-classical genomic or non-genomic actions of the ERα, or 3) ERα-independent mechanisms. Here we studied the role of EGFR signalling in this process, using estrogen responsive MCF7 cells that have increased expression of wild type EGFR. We showed that EGF-driven signalling in these cells is sufficient to maintain ERα-independent cell proliferation.

We generated a MCF7 cell line with ectopic expression of EGFR, which allowed the unbiased analysis of the interaction of EGFR and ERα signalling. In contrast, in many studies on the mechanism of tamoxifen resistance, MCF7 cells are used that already have an increased expression or constitutive activation of EGFR and/or downstream MAPK or Akt activation due to long term culture in the presence of tamoxifen [9,10,22]. This prohibits the investigation of the intrinsic effect of EGFR signalling on the antagonistic activity of tamoxifen in cells that, in the absence of EGF, respond similarly as the parental MCF7 cells. With respect to ERα expression, this was similar in our MCF7-EGFR and parent MCF7 cells, and resembles tamoxifen resistant ERα positive human tumours that express ERα at normal levels [56,57]. Therefore, our MCF7-EGFR cell line represents an important tool to study the mechanisms of tamoxifen resistance in a more clinically relevant model.

Ectopic expression of human EGFR in MCF7 cells induced cell proliferation upon stimulation with EGF, which was ERα-independent, since ERα knock down did not affect EGF induced proliferation. In agreement with this, EGF-induced proliferation was not blocked by tamoxifen or fulvestrant. Therefore, increased EGFR expression in ERα positive breast cancers may be a sole important determinant for prediction of anti-estrogen resistance. Although our data are consistent with literature data showing tamoxifen, and also (partly) fulvestrant resistance upon increased EGFR expression in breast cancer cells, typically these studies involved human breast cancer cell lines that were long term cultured in the presence of these antagonists [9,10,22]. It cannot be excluded that additional changes in other cellular signalling pathways parallel or downstream of the EGFR may be mutated in these models as well. It is relevant to note that while EGF-induced cell proliferation in MCF7-EGFR cells was ERα independent and tamoxifen insensitive, the majority of E2-induced transcriptional changes in MCF7-EGFR cells remained sensitive to tamoxifen after EGF stimulation. These data clearly indicate that during E2 and EGF co-exposure, cell proliferation and E2-induced transcription are controlled by different signalling pathways.

The parental MCF7 and MCF7-EGFR cells showed a small increase in MAPK_1/3_ activation after E2 stimulation which seems consistent with the results of Migliaccio et al. [58] who observed a 2–3 fold MAPK_1/3_ activation in MCF7 cells several minutes after estradiol exposure by measuring radiolabelled phosphate incorporation in a MAPK substrate. However, the increase MAPK_1/3_ activation by EGF in our MCF7 and MCF7-EGFR cells (5 and 35 fold respectively) is much bigger than the activation by estradiol (1.5 and 2 fold).

In tamoxifen resistant breast tumour cells an agonistic effect was observed by tamoxifen both at the level of ERα-mediated transcription and cell proliferation [9,33]. It has been suggested that these effects of tamoxifen depend on the phosphorylation of ERα by MAPK_1/3_[9,33]. However, not all groups find agonistic effects of tamoxifen on transcription and/or proliferation after increased MAPK activation and ERα serine 118 phosphorylation [34]. Similarly, in our MCF7-EGFR model also no agonistic effects of tamoxifen were observed on proliferation and transcription. This is not surprising as the proliferation of MCF7-EGFR cells after EGF stimulation is already high, and any possible additional agonistic effects of tamoxifen may therefore not become manifest. However, it may also be the result of other cell types being used in the previous studies compared to our present cell lines. The lack of an agonistic effect of tamoxifen on transcription after EGFR activation actually suggests that no agonistic effects of tamoxifen are induced in our MCF7-EGFR cells by enhanced EGFR signalling.

EGFR activation in MCF7-EGFR cells caused strong downstream activation of both the MAPK and Akt signalling cascades. Using specific inhibitors we demonstrated that the MEK/MAPK pathway is not dominant in EGFR-driven proliferation. Recently, using insertion mutagenesis in an estrogen-dependent breast carcinoma cell line, a panel of 7 candidate breast cancer anti-estrogen resistant (BCAR) genes were identified that directly underlie estrogen independence leading to tamoxifen resistance, including both EGFR, AKT1, and AKT2 [59]. Importantly, the mRNA levels of these latter candidates in breast cancer material were significantly correlated with progression or metastasis free survival [60]. These data support our findings about the importance of the PI3K/Akt in the EGFR signalling leading to estrogen independent proliferation and tamoxifen insensitivity. The remaining question is which of the downstream targets of AKT are ultimately responsible for EGF- induced proliferation in MCF7-EGFR cells. One of the candidates may be c-Jun NH2- terminal kinase (JNK), which can regulate activator protein (AP)-1 transcription of e.g. cyclin D1 and other proliferation and survival genes. This hypothesis is strengthened e.g. by the data of Johnston et al., showing increased JNK activity and AP-1 DNA-binding in tumours of resistant patients [61]. Our transcriptomic data are in agreement with this, but also show activation of other cell survival and proliferation signalling pathways by EGF, such as Smad3 and Stat3 signalling, and most likely each of these contribute to overall cell growth induced by EGFR activation in MCF7/EGFR cells.

## Conclusions

In conclusion, in this paper we have shown that ectopic expression of EGFR creates an enhanced EGFR signalling that can take over proliferation signalling when E2-driven proliferation is inhibited by anti-estrogen therapy. This EGFR-driven proliferation may be dependent on the PI3K/Akt pathway and to a lesser extent on the MEK/MAPK pathway.

To overcome anti-estrogen insensitivity induced by this EGFR signalling, treatment with inhibitors of the EGFR-PI3K/Akt signalling pathway is indicated. However, our model shows that EGFR over expressing cells may still be estrogen sensitive after such treatment. Therefore, EGFR-PI3K/Akt pathway inhibitors should preferentially be combined with anti- estrogen treatment.

## Abbreviations

ERα: Estrogen receptor alpha; EGF (R): Epidermal growth factor (receptor); E2: 17β-estradiol; PI3K: Phosphoinositide 3-kinase; Akt: v-akt murine thymoma viral oncogene homolog; MEK: Mitogen activated protein kinase kinase; MAPK1/3: Mitogen activated protein kinase 1/3; RTK: Receptor tyrosine kinase; TAM: 4-hydroxy-tamoxifen; SRB: Sulforhodamin B; siRNA: Short interference RNA; ERE: Estrogen responsive element.

## Competing interests

The authors declare that they have no competing interests.

## Authors’ contributions

MM determined MAPK and Akt activation, performed the SRB proliferation assays, the siRNA knock-down experiments, the luciferase assays and drafted the manuscript. YZ established the MCF7-EGFR cells and characterized these by immunofluorescence and immunoblotting. LW performed the Hoechst cell proliferation assay. JM and BvdW conceived of the study and designed and coordinated the experiments and helped to draft the manuscript. All authors read and approved the final manuscript.

## Pre-publication history

The pre-publication history for this paper can be accessed here:

http://www.biomedcentral.com/1471-2407/14/283/prepub

## Supplementary Material

Additional file 1: Figure S1 EGFR over expression induces tamoxifen resistance as measured by an alternative cell proliferation assay. MCF7-EGFR cells were estrogen starved 48 hours prior to a 5 day proliferation period in the absence or presence of 0.1 nM E2 with or without EGF (100 ng/mL) and a concentration series of TAM. Afterwards, cells were treated and stained with Hoechst 33258 as described in the Methods section. Data represent the average ± SEM (n = 3). Click here for file

Additional file 2: Figure S2 EGF does not downregulate ERα. After 48 hours estrogen starvation, MCF7-wt and MCF7-EGFR cells were exposed to 100 ng/mL EGF at day 1 and 3, and EGFR and ERα were analysed on western blots after 2 and 5 days. The ratios of EGFR and ERα over tubulin are indicated below the blots. Click here for file

Additional file 3: Figure S3 Ectopic EGFR expression does not induce tamoxifen resistance on the transcriptional level. Parental MCF7 **(A)** and MCF7-EGFR **(B)** cells were transiently transfected with an ERE-tk- luciferase construct and estrogen starved for 48 hours before stimulation with either E2 (0.1 nM) or EGF (100 ng/mL), with or without TAM (100 nM), or with E2, EGF and TAM, for 12 hours. The normalised luminescence intensity is shown. Click here for file

Additional file 4: Figure S4 EGF stimulation of MCF7-EGFR cells induces only little ERE-dependent transcription that is not enhanced by TAM. Parental MCF7 **(A)** and MCF7-EGFR **(B)** cells were transiently transfected with an ERE-tk- luciferase construct and estrogen starved for 48 hours before stimulation with either E2 (0.1 nM) or EGF (100 ng/mL), with or without TAM (100 nM), for 2–12 hours. The normalised luminescence intensity is shown. Click here for file

Additional file 5: Figure S5 Ectopic EGFR expression does not induce agonistic effects of tamoxifen. Parental MCF7 and MCF7-EGFR cells were estrogen starved 48 hours prior to a 5 day proliferation period with a concentration series TAM, with or without 100 ng/mL EGF. Afterwards, cells were fixed with 50% TCA and stained with sulforhodamin B, which absorbance was measured at 540 nm. Data represent the average ± SEM (n = 3). Click here for file

Additional file 6: Table S1 Agonistic effect of E2 and EGF on gene expression. Click here for file

Additional file 7: Table S2 Antagonistic effect of EGF on E2 induced gene expression. Click here for file

Additional file 8: Table S3 E2 and EGF induced changes in gene expression related to cell proliferation. Click here for file
