# Supplementary figures and images for: Epidermal growth factor receptor signalling in human breast cancer cells operates parallel to estrogen receptor α signalling and results in tamoxifen insensitive proliferation
Source: BMC Cancer. 2014 Apr 23;14:283. doi: 10.1186/1471-2407-14-283 (PMC4021213; doi:10.1186/1471-2407-14-283)

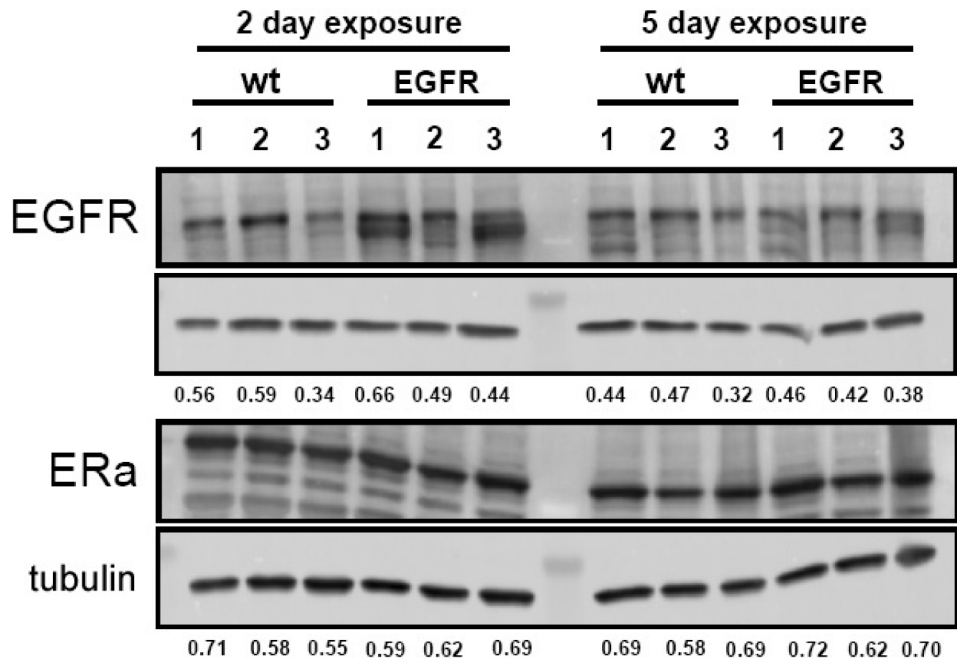

1. Starvation without EGF
2. Starvation with EGF
3. Basal medium

Supplement: Additional file 1: Figure S1 — EGFR over expression induces tamoxifen resistance as measured by an alternative cell proliferation assay. MCF7-EGFR cells were estrogen starved 48 hours prior to a 5 day proliferation period in the absence or presence of 0.1 nM E2 with or without EGF (100 ng/mL) and a concentration series of TAM. Afterwards, cells were treated and stained with Hoechst 33258 as described in the Methods section. Data represent the average ± SEM (n = 3). [file 1471-2407-14-283-S1.pdf]

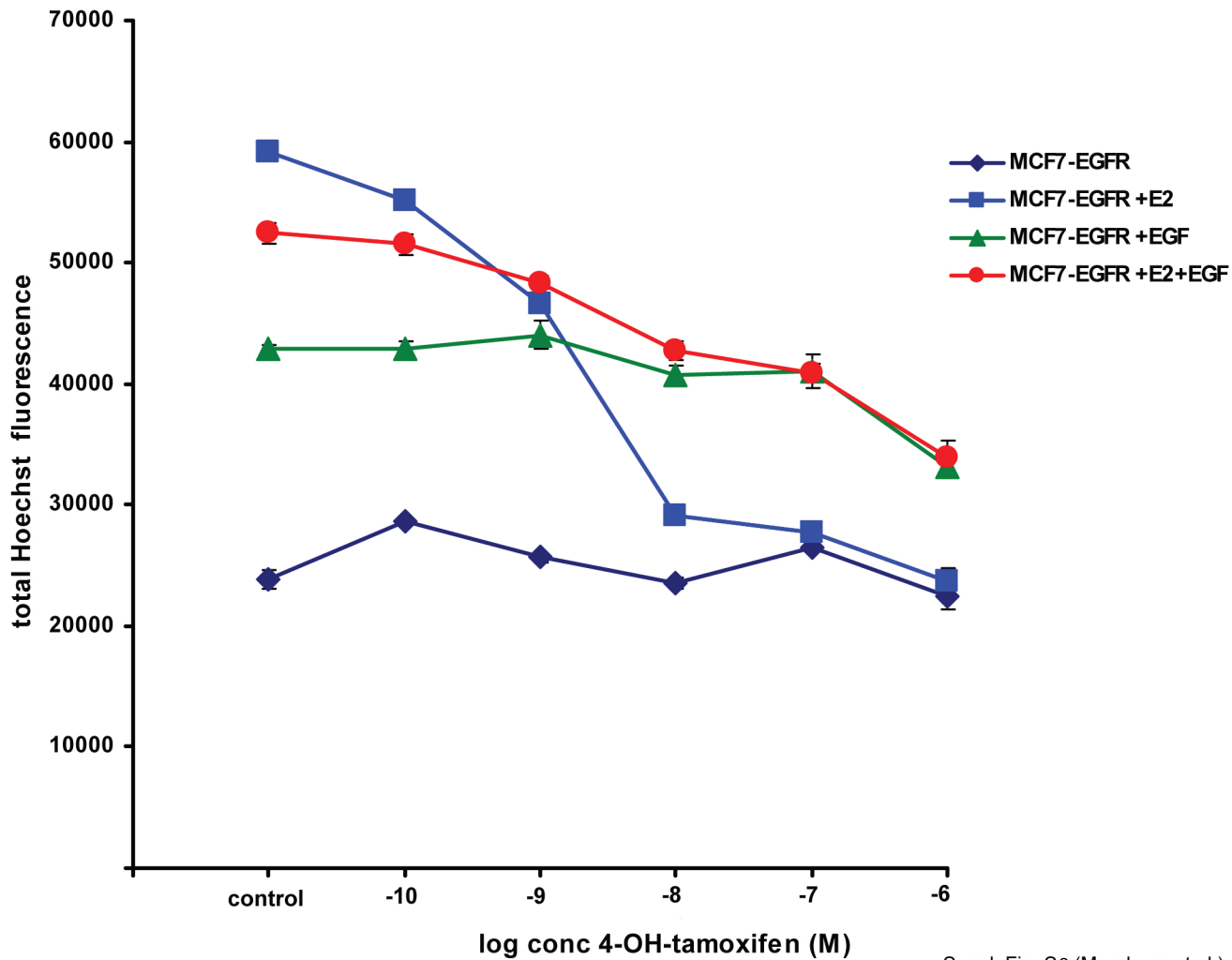

Supplement: Additional file 2: Figure S2 — EGF does not downregulate ERα. After 48 hours estrogen starvation, MCF7-wt and MCF7-EGFR cells were exposed to 100 ng/mL EGF at day 1 and 3, and EGFR and ERα were analysed on western blots after 2 and 5 days. The ratios of EGFR and ERα over tubulin are indicated below the blots. [file 1471-2407-14-283-S2.pdf]

**A.**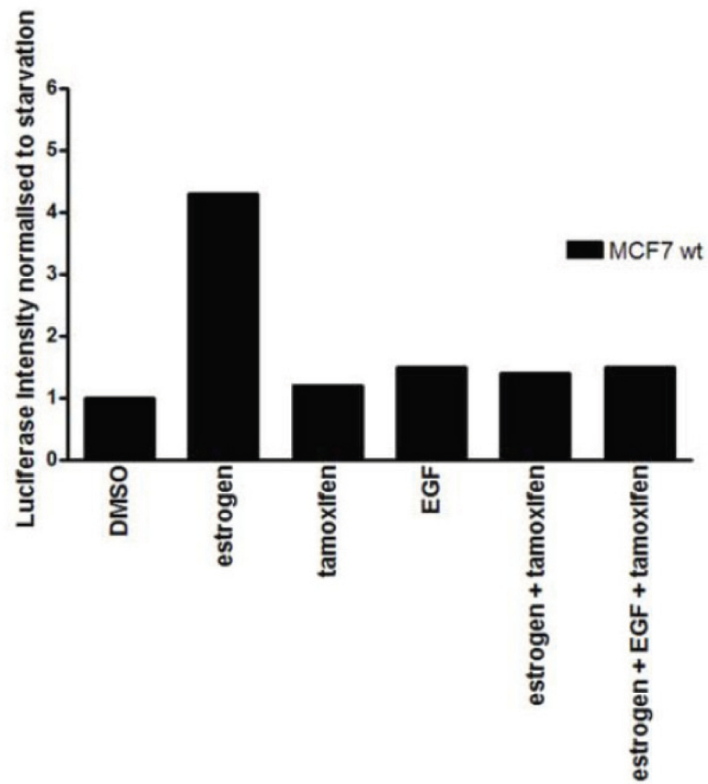**B.**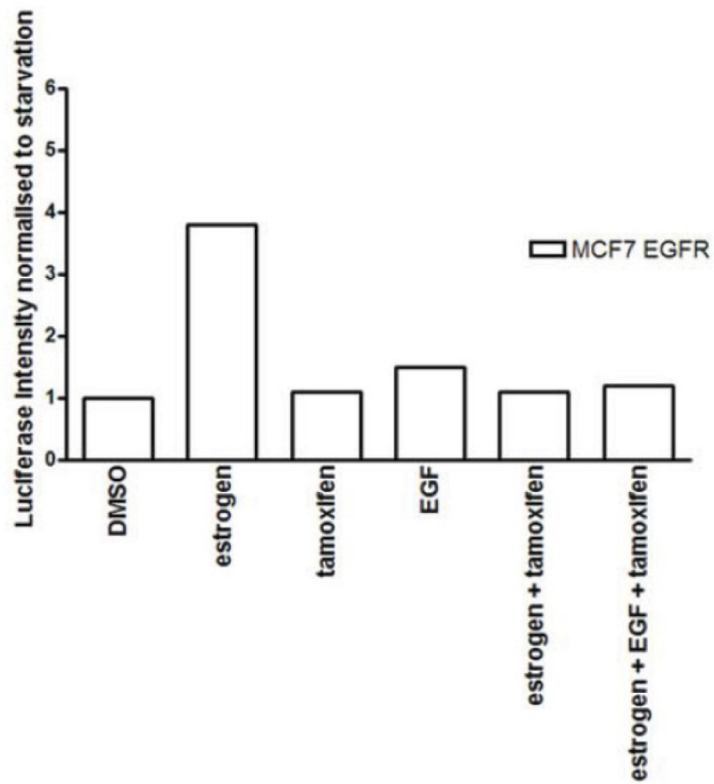

Supplement: Additional file 3: Figure S3 — Ectopic EGFR expression does not induce tamoxifen resistance on the transcriptional level. Parental MCF7 (A) and MCF7-EGFR (B) cells were transiently transfected with an ERE-tk- luciferase construct and estrogen starved for 48 hours before stimulation with either E2 (0.1 nM) or EGF (100 ng/mL), with or without TAM (100 nM), or with E2, EGF and TAM, for 12 hours. The normalised luminescence intensity is shown. [file 1471-2407-14-283-S3.pdf]

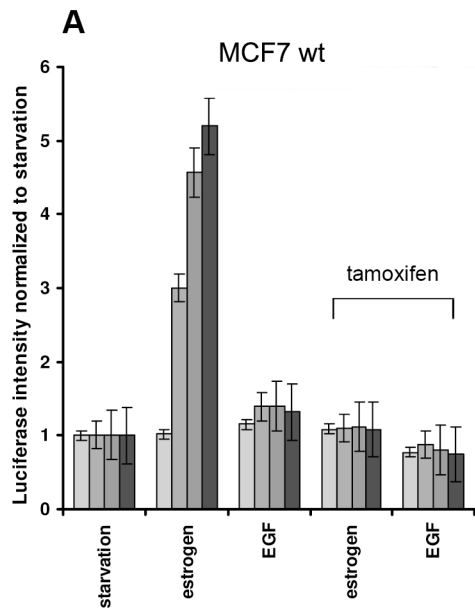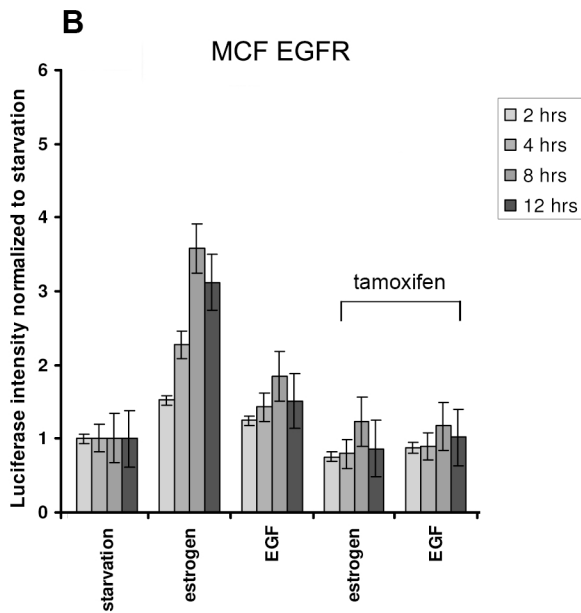

Supplement: Additional file 4: Figure S4 — EGF stimulation of MCF7-EGFR cells induces only little ERE-dependent transcription that is not enhanced by TAM. Parental MCF7 (A) and MCF7-EGFR (B) cells were transiently transfected with an ERE-tk- luciferase construct and estrogen starved for 48 hours before stimulation with either E2 (0.1 nM) or EGF (100 ng/mL), with or without TAM (100 nM), for 2–12 hours. The normalised luminescence intensity is shown. [file 1471-2407-14-283-S4.pdf]

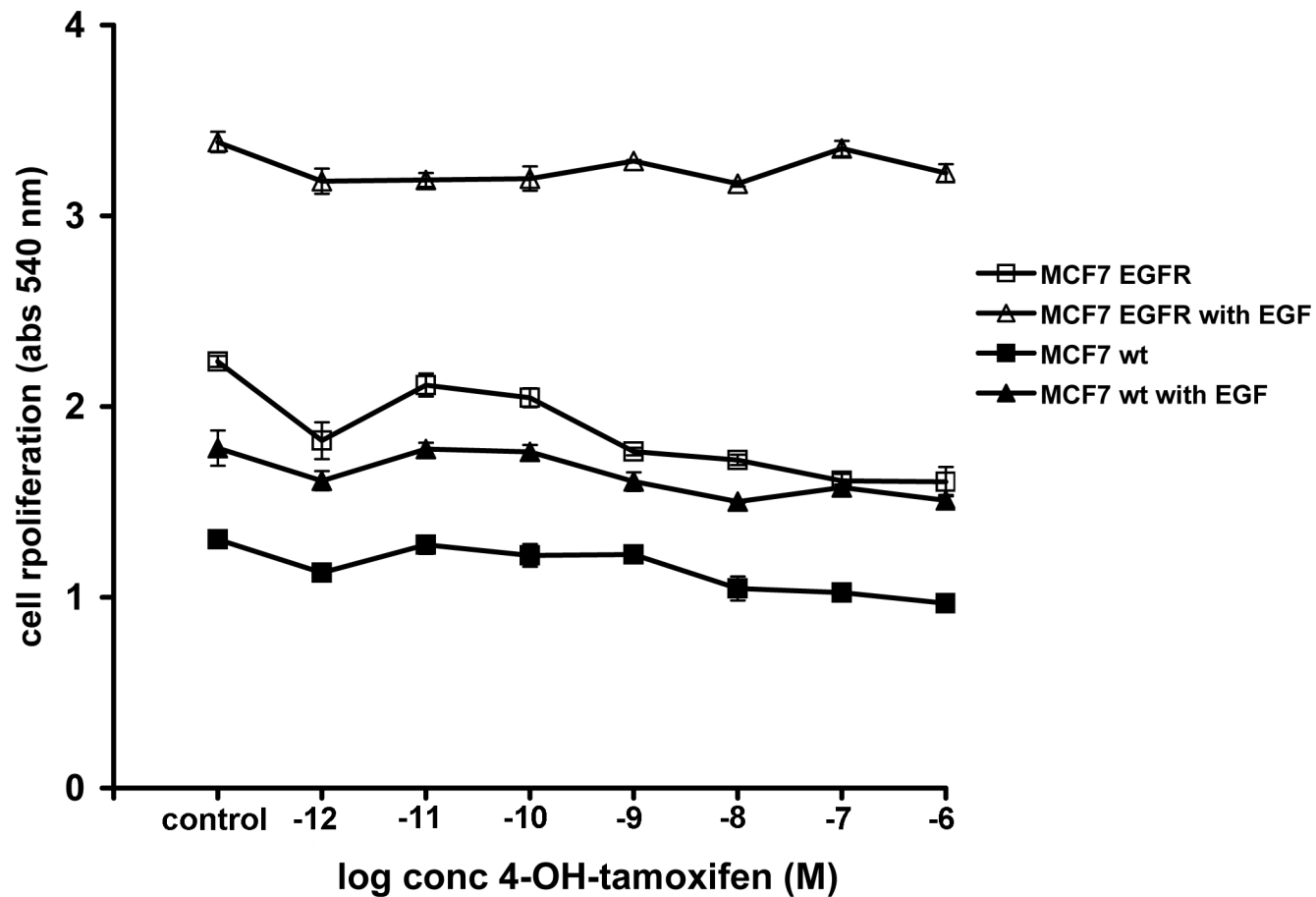

Supplementary Figure S5 (Moerkens et al.)

Supplement: Additional file 5: Figure S5 — Ectopic EGFR expression does not induce agonistic effects of tamoxifen. Parental MCF7 and MCF7-EGFR cells were estrogen starved 48 hours prior to a 5 day proliferation period with a concentration series TAM, with or without 100 ng/mL EGF. Afterwards, cells were fixed with 50% TCA and stained with sulforhodamin B, which absorbance was measured at 540 nm. Data represent the average ± SEM (n = 3). [file 1471-2407-14-283-S5.pdf]
