# Supplementary material for: Epidermal growth factor receptor signalling in human breast cancer cells operates parallel to estrogen receptor α signalling and results in tamoxifen insensitive proliferation
Source: BMC Cancer. 2014 Apr 23;14:283. doi: 10.1186/1471-2407-14-283 (PMC4021213; doi:10.1186/1471-2407-14-283)
Supplement: Additional file 6: Table S1 — Agonistic effect of E2 and EGF on gene expression. [file 1471-2407-14-283-S6.doc]

| **Additional Table S1*. Agonistic effect of E2 and EGF on gene expression*** | | |  |  |  |  |  |  |  |  |  |  |  |
| --- | --- | --- | --- | --- | --- | --- | --- | --- | --- | --- | --- | --- | --- |
| **Upregulated genes** | |  |  |  |  |  |  |  |  |  |  |  |  |
| **Gene symbol** | **EntrezID** | **Description** | **E2** | **control** | **Fold-change** | | **EGF** | **control** | **Fold-change** | | **E2+EGF** | **control** | **Fold-change** |
| ABCE1 | 6059 | ATP-binding cassette, sub-family E (OABP), member 1 | 880.19 | 550.18 | **1.6** |  | 834.33 | 550.18 | **1.52** |  | 1054.24 | 550.18 | **1.92** |
| ABHD2 | 11057 | abhydrolase domain containing 2 | 108.96 | 72.84 | **1.5** |  | 140.71 | 72.84 | **1.93** |  | 216.92 | 72.84 | **2.98** |
| ADCY3 | 109 | adenylate cyclase 3 | 80.44 | 51.2 | **1.57** |  | 87.32 | 51.2 | **1.71** |  | 141.01 | 51.2 | **2.75** |
| AMD1 | 262 | adenosylmethionine decarboxylase 1 | 557.86 | 306.69 | **1.82** |  | 513.79 | 306.69 | **1.68** |  | 696.05 | 306.69 | **2.27** |
| ANKH | 56172 | ankylosis, progressive homolog (mouse) | 203.4 | 104.89 | **1.94** |  | 233.3 | 104.89 | **2.22** |  | 563.46 | 104.89 | **5.37** |
| ARHGAP26 | 23092 | Rho GTPase activating protein 26 | 31.04 | 20.41 | **1.52** |  | 38.75 | 20.41 | **1.9** |  | 77.28 | 20.41 | **3.79** |
| BAG2 | 9532 | BCL2-associated athanogene 2 | 109.23 | 60.05 | **1.82** |  | 121.13 | 60.05 | **2.02** |  | 168.35 | 60.05 | **2.8** |
| CEBPG | 1054 | CCAAT/enhancer binding protein (C/EBP), gamma | 226.83 | 146.6 | **1.55** |  | 295.68 | 146.6 | **2.02** |  | 400.56 | 146.6 | **2.73** |
| CNKSR3 | 154043 | CNKSR family member 3 | 83.89 | 40.45 | **2.07** |  | 91.97 | 40.45 | **2.27** |  | 131.86 | 40.45 | **3.26** |
| CTSD | 1509 | cathepsin D | 54.53 | 13.28 | **4.11** |  | 32.62 | 13.28 | **2.46** |  | 118.11 | 13.28 | **8.9** |
| DAPK2 | 23604 | death-associated protein kinase 2 | 46.66 | 26.83 | **1.74** |  | 64.03 | 26.83 | **2.39** |  | 132.05 | 26.83 | **4.92** |
| DNAJC12 | 56521 | DnaJ (Hsp40) homolog, subfamily C, member 12 | 179.05 | 117.26 | **1.53** |  | 274.81 | 117.26 | **2.34** |  | 368.35 | 117.26 | **3.14** |
| DOK7 | 285489 | docking protein 7 | 581.15 | 245.34 | **2.37** |  | 373.57 | 245.34 | **1.52** |  | 806.92 | 245.34 | **3.29** |
| DYNLT3 | 6990 | dynein, light chain, Tctex-type 3 | 1590.58 | 873.68 | **1.82** |  | 1575.97 | 873.68 | **1.8** |  | 2055.83 | 873.68 | **2.35** |
| EGR3 | 1960 | early growth response 3 | 1008.41 | 171.92 | **5.87** |  | 919.4 | 171.92 | **5.35** |  | 2076.75 | 171.92 | **12.08** |
| FAIM3 | 9214 | Fas apoptotic inhibitory molecule 3 | 198.47 | 79.07 | **2.51** |  | 137.66 | 79.07 | **1.74** |  | 295.35 | 79.07 | **3.74** |
| FHL2 | 2274 | four and a half LIM domains 2 | 879.65 | 102.06 | **8.62** |  | 704.25 | 102.06 | **6.9** |  | 1539.89 | 102.06 | **15.09** |
| FRK | 2444 | fyn-related kinase | 28.04 | 10.56 | **2.66** |  | 24.17 | 10.56 | **2.29** |  | 53.71 | 10.56 | **5.09** |
| GAD1 | 2571 | glutamate decarboxylase 1 (brain, 67kDa) | 33.3 | 18.88 | **1.76** |  | 86.67 | 18.88 | **4.59** |  | 134.59 | 18.88 | **7.13** |
| GFRA2 | 2675 | GDNF family receptor alpha 2 | 9.61 | 5.27 | **1.82** |  | 12.48 | 5.27 | **2.37** |  | 58.22 | 5.27 | **11.05** |
| GOLSYN | 55638 | Golgi-localized protein | 183.66 | 63.28 | **2.9** |  | 188.38 | 63.28 | **2.98** |  | 330.2 | 63.28 | **5.22** |
| HEG1 | 57493 | HEG homolog 1 (zebrafish) | 14.12 | 8.23 | **1.71** |  | 14.61 | 8.23 | **1.77** |  | 40.46 | 8.23 | **4.91** |
| HIPK2 | 28996 | homeodomain interacting protein kinase 2 | 810.79 | 525.04 | **1.54** |  | 917.9 | 525.04 | **1.75** |  | 1172.62 | 525.04 | **2.23** |
| IL15RA | 3601 | interleukin 15 receptor, alpha | 36.37 | 24.23 | **1.5** |  | 45.58 | 24.23 | **1.88** |  | 65.21 | 24.23 | **2.69** |
| IL20 | 50604 | interleukin 20 | 1034.85 | 252.04 | **4.11** |  | 555.57 | 252.04 | **2.2** |  | 1741.57 | 252.04 | **6.91** |
| IL24 | 11009 | interleukin 24 | 164.27 | 25.73 | **6.38** |  | 70.98 | 25.73 | **2.76** |  | 378.06 | 25.73 | **14.69** |
| IRS1 | 3667 | Insulin receptor substrate 1 | 29.98 | 17.87 | **1.68** |  | 31.56 | 17.87 | **1.77** |  | 46.04 | 17.87 | **2.58** |
| ISG20 | 3669 | interferon stimulated exonuclease gene 20kDa | 135.03 | 53.63 | **2.52** |  | 429.53 | 53.63 | **8.01** |  | 1136.15 | 53.63 | **21.18** |
| ISG20 | 3669 | interferon stimulated exonuclease gene 20kDa | 255.78 | 97.24 | **2.63** |  | 798.53 | 97.24 | **8.21** |  | 2015.46 | 97.24 | **20.73** |
| LOC388796 | 388796 | hypothetical LOC388796 | 173.71 | 105.44 | **1.65** |  | 166.84 | 105.44 | **1.58** |  | 234.14 | 105.44 | **2.22** |
| LOC402778 | 402778 | similar to RIKEN cDNA 6330512M04 gene (mouse) | 34.79 | 14.45 | **2.41** |  | 26.32 | 14.45 | **1.82** |  | 90.23 | 14.45 | **6.24** |
| LONRF2 | 164832 | LON peptidase N-terminal domain and ring finger 2 | 457.81 | 175.66 | **2.61** |  | 464.45 | 175.66 | **2.64** |  | 762.99 | 175.66 | **4.34** |
| LOXL1 | 4016 | lysyl oxidase-like 1 | 33.93 | 19.01 | **1.78** |  | 65.16 | 19.01 | **3.43** |  | 89.07 | 19.01 | **4.68** |
| MACF1 | 23499 | microtubule-actin crosslinking factor 1 | 270.22 | 167.59 | **1.61** |  | 282.32 | 167.59 | **1.68** |  | 351.1 | 167.59 | **2.1** |
| MPP6 | 51678 | membrane protein, palmitoylated 6 (MAGUK p55 subfamily member 6) | 85.67 | 49.9 | **1.72** |  | 92.46 | 49.9 | **1.85** |  | 126.73 | 49.9 | **2.54** |
| MREG | 55686 | melanoregulin | 548.95 | 256.64 | **2.14** |  | 529.91 | 256.64 | **2.06** |  | 754.05 | 256.64 | **2.94** |
| MTHFD1L | 25902 | methylenetetrahydrofolate dehydrogenase (NADP+ dependent) 1-like | 859.23 | 498.17 | **1.72** |  | 963.39 | 498.17 | **1.93** |  | 1231.9 | 498.17 | **2.47** |
| NAV2 | 89797 | neuron navigator 2 | 106.68 | 29.69 | **3.59** |  | 162.8 | 29.69 | **5.48** |  | 238.26 | 29.69 | **8.03** |
| NBPF22P | 148545 | neuroblastoma breakpoint family, member 22 (pseudogene) | 185.22 | 69.34 | **2.67** |  | 152.27 | 69.34 | **2.2** |  | 351.9 | 69.34 | **5.07** |
| NOLC1 | 9221 | nucleolar and coiled-body phosphoprotein 1 | 327.61 | 212.31 | **1.54** |  | 360.21 | 212.31 | **1.7** |  | 448.99 | 212.31 | **2.11** |
| NP | 4860 | nucleoside phosphorylase | 508.18 | 294.07 | **1.73** |  | 467.48 | 294.07 | **1.59** |  | 649.6 | 294.07 | **2.21** |
| PADI1 | 29943 | peptidyl arginine deiminase, type I | 14.36 | 9.15 | **1.57** |  | 47.65 | 9.15 | **5.21** |  | 98.96 | 9.15 | **10.81** |
| PADI3 | 51702 | peptidyl arginine deiminase, type III | 153.45 | 18.78 | **8.17** |  | 38.18 | 18.78 | **2.03** |  | 238.89 | 18.78 | **12.72** |
| PCOLCE2 | 26577 | procollagen C-endopeptidase enhancer 2 | 17.33 | 10.94 | **1.58** |  | 24.47 | 10.94 | **2.24** |  | 36.03 | 10.94 | **3.29** |
| PLAC1 | 10761 | placenta-specific 1 | 89.61 | 29.74 | **3.01** |  | 83.6 | 29.74 | **2.81** |  | 212.22 | 29.74 | **7.14** |
| PMAIP1 | 5366 | phorbol-12-myristate-13-acetate-induced protein 1 | 199.78 | 64.66 | **3.09** |  | 177.13 | 64.66 | **2.74** |  | 289.44 | 64.66 | **4.48** |
| PPM1E | 22843 | protein phosphatase 1E (PP2C domain containing) | 33.34 | 18.38 | **1.81** |  | 29.73 | 18.38 | **1.62** |  | 45.97 | 18.38 | **2.5** |
| PRAGMIN | 157285 | homolog of rat pragma of Rnd2 | 953.15 | 292.45 | **3.26** |  | 654.12 | 292.45 | **2.24** |  | 1493.17 | 292.45 | **5.11** |
| PRSS23 | 11098 | protease, serine, 23 | 314.96 | 101.38 | **3.11** |  | 379.28 | 101.38 | **3.74** |  | 619.66 | 101.38 | **6.11** |
| PSAT1 | 29968 | phosphoserine aminotransferase 1 | 455.92 | 220.91 | **2.06** |  | 672.57 | 220.91 | **3.04** |  | 951.01 | 220.91 | **4.3** |
| RAB31 | 11031 | RAB31, member RAS oncogene family | 1555.43 | 547.23 | **2.84** |  | 1885.88 | 547.23 | **3.45** |  | 3481.5 | 547.23 | **6.36** |
| RAI14 | 26064 | retinoic acid induced 14 | 113.8 | 52.71 | **2.16** |  | 109.1 | 52.71 | **2.07** |  | 219.79 | 52.71 | **4.17** |
| RUNX2 | 860 | runt-related transcription factor 2 | 50.99 | 23.71 | **2.15** |  | 124.01 | 23.71 | **5.23** |  | 178.95 | 23.71 | **7.55** |
| SEMA3B | 7869 | sema domain, immunoglobulin domain (Ig), short basic domain, secreted, (semaphorin) 3B | 26.22 | 14.8 | **1.77** |  | 27.98 | 14.8 | **1.89** |  | 102.11 | 14.8 | **6.9** |
| SLC16A1 | 6566 | solute carrier family 16, member 1 (monocarboxylic acid transporter 1) | 47.46 | 24.4 | **1.94** |  | 54.47 | 24.4 | **2.23** |  | 70.15 | 24.4 | **2.87** |
| SLC1A4 | 6509 | solute carrier family 1 (glutamate/neutral amino acid transporter), member 4 | 237.1 | 93.09 | **2.55** |  | 178.18 | 93.09 | **1.91** |  | 407.89 | 93.09 | **4.38** |
| SLC3A2 | 6520 | solute carrier family 3 (activators of dibasic and neutral amino acid transport), member 2 | 675.34 | 340.33 | **1.98** |  | 579.55 | 340.33 | **1.7** |  | 936.61 | 340.33 | **2.75** |
| SOBP | 55084 | sine oculis binding protein homolog (Drosophila) | 26.47 | 16.82 | **1.57** |  | 25.87 | 16.82 | **1.54** |  | 31.79 | 16.82 | **1.89** |
| SVIL | 6840 | supervillin | 207.28 | 81.49 | **2.54** |  | 151.08 | 81.49 | **1.85** |  | 306.93 | 81.49 | **3.77** |
| SYTL5 | 94122 | synaptotagmin-like 5 | 2575.55 | 930.81 | **2.77** |  | 2606.8 | 930.81 | **2.8** |  | 3945.09 | 930.81 | **4.24** |
| TAF5L | 27097 | TAF5-like RNA polymerase II, p300/CBP-associated factor (PCAF)-associated factor, 65kDa | 99.3 | 65.55 | **1.51** |  | 103.25 | 65.55 | **1.58** |  | 132.42 | 65.55 | **2.02** |
| THBS1 | 7057 | Thrombospondin 1 | 122.54 | 40.73 | **3.01** |  | 113.42 | 40.73 | **2.78** |  | 186.02 | 40.73 | **4.57** |
| TIAM1 | 7074 | T-cell lymphoma invasion and metastasis 1 | 264.93 | 126.05 | **2.1** |  | 221.63 | 126.05 | **1.76** |  | 386.29 | 126.05 | **3.06** |
| TMEM2 | 23670 | transmembrane protein 2 | 557.4 | 312.12 | **1.79** |  | 593.22 | 312.12 | **1.9** |  | 799.85 | 312.12 | **2.56** |
| TMPRSS3 | 64699 | transmembrane protease, serine 3 | 229.76 | 34.38 | **6.68** |  | 99.35 | 34.38 | **2.89** |  | 671.67 | 34.38 | **19.54** |
| TPM1 | 7168 | tropomyosin 1 (alpha) | 2309.87 | 1122.05 | **2.06** |  | 1930.02 | 1122.05 | **1.72** |  | 3050.6 | 1122.05 | **2.72** |
| TRAF3 | 7187 | TNF receptor-associated factor 3 | 430.86 | 276.29 | **1.56** |  | 440.38 | 276.29 | **1.59** |  | 540.91 | 276.29 | **1.96** |
| TSPAN5 | 10098 | tetraspanin 5 | 17.05 | 10.33 | **1.65** |  | 70.26 | 10.33 | **6.8** |  | 103.81 | 10.33 | **10.05** |
|  |  | **average** |  |  | **2.51** |  |  |  | **2.67** |  |  |  | **5.57** |
| **Downregulated genes** | |  |  |  |  |  |  |  |  |  |  |  |  |
| **Gene symbol** | **EntrezID** | **Description** | **E2** | **control** | **Fold-change** | | **EGF** | **control** | **Fold-change** | | **E2+EGF** | **control** | **Fold-change** |
| UTRN | 7402 | utrophin | 151.53 | 234.07 | **-1.54** |  | 151.43 | 234.07 | **-1.54** |  | 88.58 | 234.07 | **-2.63** |
| TMPRSS2 | 7113 | transmembrane protease, serine 2 | 17.14 | 27.33 | **-1.59** |  | 16.41 | 27.33 | **-1.67** |  | 11.33 | 27.33 | **-2.44** |
| SYT1 | 6857 | synaptotagmin I | 255.99 | 387.13 | **-1.52** |  | 248.04 | 387.13 | **-1.56** |  | 169.83 | 387.13 | **-2.27** |
| STAU2 | 27067 | staufen, RNA binding protein, homolog 2 (Drosophila) | 69.36 | 107.65 | **-1.56** |  | 53.25 | 107.65 | **-2.04** |  | 25.26 | 107.65 | **-4.35** |
| SPATA17 | 128153 | spermatogenesis associated 17 | 45.7 | 69.27 | **-1.52** |  | 40.43 | 69.27 | **-1.72** |  | 30.6 | 69.27 | **-2.27** |
| SLC31A2 | 1318 | solute carrier family 31 (copper transporters), member 2 | 61.05 | 94.45 | **-1.54** |  | 54.6 | 94.45 | **-1.72** |  | 43.62 | 94.45 | **-2.17** |
| PPARA | 5465 | peroxisome proliferator-activated receptor alpha | 39.93 | 61.64 | **-1.54** |  | 29.13 | 61.64 | **-2.13** |  | 20.95 | 61.64 | **-2.94** |
| PACSIN1 | 29993 | protein kinase C and casein kinase substrate in neurons 1 | 38.43 | 58.69 | **-1.54** |  | 37.28 | 58.69 | **-1.56** |  | 26.65 | 58.69 | **-2.22** |
| IGSF5 | 150084 | immunoglobulin superfamily, member 5 | 18.54 | 30.04 | **-1.61** |  | 18.17 | 30.04 | **-1.67** |  | 12.54 | 30.04 | **-2.38** |
| FXYD3 | 5349 | FXYD domain containing ion transport regulator 3 | 181.07 | 275.98 | **-1.52** |  | 163.07 | 275.98 | **-1.69** |  | 126.19 | 275.98 | **-2.17** |
| ERBB3 | 2065 | v-erb-b2 erythroblastic leukemia viral oncogene homolog 3 (avian) | 1018.09 | 1545.86 | **-1.52** |  | 909.91 | 1545.86 | **-1.69** |  | 727.92 | 1545.86 | **-2.13** |
| ELF5 | 2001 | E74-like factor 5 (ets domain transcription factor) | 52.83 | 88.65 | **-1.67** |  | 32.13 | 88.65 | **-2.78** |  | 19.18 | 88.65 | **-4.55** |
| DZIP3 | 9666 | DAZ interacting protein 3, zinc finger | 107.3 | 162.68 | **-1.52** |  | 98.31 | 162.68 | **-1.67** |  | 68.65 | 162.68 | **-2.38** |
| CLMN | 79789 | calmin (calponin-like, transmembrane) | 108.87 | 226.45 | **-2.08** |  | 98.59 | 226.45 | **-2.27** |  | 45.21 | 226.45 | **-5.00** |
| CGN | 57530 | cingulin | 152.42 | 262.91 | **-1.72** |  | 117.13 | 262.91 | **-2.22** |  | 80.44 | 262.91 | **-3.23** |
| CAB39L | 81617 | calcium binding protein 39-like | 106.16 | 213.95 | **-2.00** |  | 67.27 | 213.95 | **-3.23** |  | 36.84 | 213.95 | **-5.88** |
| C2orf67 | 151050 | chromosome 2 open reading frame 67 | 15.3 | 23.67 | **-1.54** |  | 15.06 | 23.67 | **-1.56** |  | 10.48 | 23.67 | **-2.27** |
| BCL2L1 | 598 | BCL2-like 1 | 83.18 | 127.54 | **-1.54** |  | 73.28 | 127.54 | **-1.75** |  | 56.35 | 127.54 | **-2.27** |
| ARHGEF6 | 9459 | Rac/Cdc42 guanine nucleotide exchange factor (GEF) 6 | 55.82 | 90.9 | **-1.64** |  | 56.01 | 90.9 | **-1.61** |  | 35.64 | 90.9 | **-2.56** |
| ANK3 | 288 | ankyrin 3, node of Ranvier (ankyrin G) | 747.84 | 1179.64 | **-1.59** |  | 775.09 | 1179.64 | **-1.52** |  | 521.02 | 1179.64 | **-2.27** |
| ADCY6 | 112 | adenylate cyclase 6 | 44.49 | 69.48 | **-1.56** |  | 36.99 | 69.48 | **-1.89** |  | 28.25 | 69.48 | **-2.44** |
| ABCB1 | 5243 | ATP-binding cassette, sub-family B (MDR/TAP), member 1 | 46.02 | 85.7 | **-1.85** |  | 35.5 | 85.7 | **-2.44** |  | 23.11 | 85.7 | **-3.70** |
|  |  | **average** |  |  | **-1.62** |  |  |  | **-1.91** |  |  |  | **-2.93** |
|  |  |  |  |  |  |  |  |  |  |  |  |  |  |
|  |  |  |  |  |  |  |  |  |  |  |  |  |  |
|  |  |  |  |  |  |  |  |  |  |  |  |  |  |
| **Transcription Factors identified by Metacore software and their targets** | | | **AR** | **c-JUN** | **c-MYC** | **EGR1** | **ESR1** | **HIF1A** | **p53** | **SP1** |  |  |  |
|  |  |  | ABCE1 | ERBB3 | NOLC1 | NOLC1 | IRS1 | IL20 | ISG20 | IRS1 |  |  |  |
|  |  |  | ARHGAP26 | SLC3A2 | THBS1 | ABHD2 | PRSS23 | PCOLCE2 | ERBB3 | NAV2 |  |  |  |
|  |  |  | TMPRSS2 | BCL2L1 | MPP6 | THBS1 | EGR3 | PMAIP1 (NOXA) | FXYD3 | BCL2L1 |  |  |  |
|  |  |  | PLAC1 | UTRN | UTRN | NAV2 | BCL2L1 | BCL2L1 | PADI3 | PADI3 |  |  |  |
|  |  |  | BCL2L1 | PSAT1 | MPP6 | PSAT1 | GOLSYN | PPARA | SLC3A2 | PADI1 |  |  |  |
|  |  |  | PMAIP1 | PADI3 | EGR3 | EGR3 | CAB39L | ANKH | MTHFD1L | THBS1 |  |  |  |
|  |  |  | THBS1 | IL24 | SLC1A4 | SYT1 | IL20 | SLC16A1 | SEMA3B | ISG20 |  |  |  |
|  |  |  | TMEM2 | THBS1 | ISG20 | GAD1 | RUNX2 | CTSD | ABCB1 | FHL2 |  |  |  |
|  |  |  | FXYD3 | ABCB1 | PMAIP1 | ABCB1 | PADI3 | ABCB1 | FHL2 | SLC16A1 |  |  |  |
|  |  |  | AMD1 |  | AMD1 |  | PRAGMIN |  | PMAIP1 | IL24 |  |  |  |
|  |  |  |  |  | MTHFD1L |  | THBS1 |  | CTSD | GAD1 |  |  |  |
|  |  |  |  |  | ADCY3 |  | IL24 |  | THBS1 | PLAC1 |  |  |  |
|  |  |  |  |  | NP |  | CGN |  |  | TPM1 |  |  |  |
|  |  |  |  |  | DNAJC12 |  | DNAJC12 |  |  | DAPK2 |  |  |  |
|  |  |  |  |  | DAPK2 |  | PPM1E |  |  | RUNX2 |  |  |  |
|  |  |  |  |  | TPM1 |  | PPARA |  |  | UTRN |  |  |  |
|  |  |  |  |  | ABCE1 |  | HEG1 |  |  | ABCB1 |  |  |  |
|  |  |  |  |  | GAD1 |  | ABCB1 |  |  | CTSD |  |  |  |
|  |  |  |  |  | SLC3A2 |  | SEMA3B |  |  | SLC3A2 |  |  |  |
|  |  |  |  |  |  |  | DYNLT3 |  |  | AMD1 |  |  |  |
|  |  |  |  |  |  |  | ANK3 |  |  | PPARA |  |  |  |
|  |  |  |  |  |  |  | FAIM3 |  |  | TMPRSS2 |  |  |  |
|  |  |  |  |  |  |  | RAB31 |  |  |  |  |  |  |
|  |  |  |  |  |  |  | ERBB3 |  |  |  |  |  |  |
|  |  |  |  |  |  |  | MREG |  |  |  |  |  |  |
|  |  |  |  |  |  |  | LONRF2 |  |  |  |  |  |  |
|  |  |  |  |  |  |  | PMAIP1 |  |  |  |  |  |  |
|  |  |  |  |  |  |  | TMPRSS3 |  |  |  |  |  |  |
|  |  |  |  |  |  |  | TMEM2 |  |  |  |  |  |  |
|  |  |  |  |  |  |  | CTSD |  |  |  |  |  |  |
|  |  |  |  |  |  |  | AMD1 |  |  |  |  |  |  |
