# Supplementary material for: Epidermal growth factor receptor signalling in human breast cancer cells operates parallel to estrogen receptor α signalling and results in tamoxifen insensitive proliferation
Source: BMC Cancer. 2014 Apr 23;14:283. doi: 10.1186/1471-2407-14-283 (PMC4021213; doi:10.1186/1471-2407-14-283)
Supplement: Additional file 7: Table S2 — Antagonistic effect of EGF on E2 induced gene expression. [file 1471-2407-14-283-S7.doc]

| **Additional Table S2*. Antagonistic effect of EGF on E2 induced gene expression*** | | |  |  |  |  |  |  |  |  |  |  |  |  |  |
| --- | --- | --- | --- | --- | --- | --- | --- | --- | --- | --- | --- | --- | --- | --- | --- |
| **E2 upregulated genes - EGF downreguletd genes** | | |  |  |  |  |  |  |  |  |  |  |  |  |  |
| **Gene symbol** | **EntrezID** | **Description** |  |  | **E2** | **control** | **Fold-change** |  | **EGF** | **control** | **Fold-change** |  | **EGF + E2** | **control** | **Fold-change** |
| ACOT6 | 641372 | acyl-CoA thioesterase 6 |  |  | 23.92 | 14.37 | **1.66** |  | 6.2 | 14.37 | **-2.32** |  | 6.05 | 14.37 | **0.42** |
| ADRB1 | 153 | adrenergic, beta-1-, receptor |  |  | 42.47 | 12 | **3.54** |  | 7.29 | 12 | **-1.65** |  | 12.49 | 12 | **1.04** |
| ASCL2 | 430 | achaete-scute complex homolog 2 (Drosophila) | |  | 214.9 | 142.37 | **1.51** |  | 94.48 | 142.37 | **-1.51** |  | 145.89 | 142.37 | **1.02** |
| BCL2 | 596 | B-cell CLL/lymphoma 2 |  |  | 731.49 | 360.58 | **2.03** |  | 183.95 | 360.58 | **-1.96** |  | 321.94 | 360.58 | **0.89** |
| BLM | 641 | Bloom syndrome, RecQ helicase-like |  |  | 166.28 | 106.22 | **1.57** |  | 69.71 | 106.22 | **-1.52** |  | 104.95 | 106.22 | **0.99** |
| BRCA1 | 672 | breast cancer 1, early onset |  |  | 231.78 | 149.12 | **1.55** |  | 95.95 | 149.12 | **-1.55** |  | 160.41 | 149.12 | **1.08** |
| C14orf83 | 161145 | chromosome 14 open reading frame 83 |  |  | 215.84 | 95.15 | **2.27** |  | 51.99 | 95.15 | **-1.83** |  | 102.55 | 95.15 | **1.08** |
| C20orf160 | 140706 | chromosome 20 open reading frame 160 |  |  | 63.52 | 35.1 | **1.81** |  | 19.45 | 35.1 | **-1.80** |  | 31.14 | 35.1 | **0.89** |
| CA12 | 771 | carbonic anhydrase XII |  |  | 4697.4 | 1517.8 | **3.09** |  | 912.13 | 1517.8 | **-1.66** |  | 2714.21 | 1517.8 | **1.79** |
| CLIC3 | 9022 | chloride intracellular channel 3 |  |  | 111.45 | 61.97 | **1.80** |  | 32.52 | 61.97 | **-1.91** |  | 70.84 | 61.97 | **1.14** |
| COL12A1 | 1303 | collagen, type XII, alpha 1 |  |  | 367.23 | 210.85 | **1.74** |  | 131.84 | 210.85 | **-1.60** |  | 138.62 | 210.85 | **0.66** |
| CPXM2 | 119587 | carboxypeptidase X (M14 family), member 2 | |  | 36.03 | 21.9 | **1.65** |  | 14.06 | 21.9 | **-1.56** |  | 25.69 | 21.9 | **1.17** |
| DLC1 | 10395 | deleted in liver cancer 1 |  |  | 547.46 | 364.02 | **1.50** |  | 241.23 | 364.02 | **-1.51** |  | 453.73 | 364.02 | **1.25** |
| DSN1 | 79980 | DSN1, MIND kinetochore complex component, homolog (S. cerevisiae) | | | 179.78 | 118.97 | **1.51** |  | 66.44 | 118.97 | **-1.79** |  | 115.49 | 118.97 | **0.97** |
| FAM111B | 374393 | family with sequence similarity 111, member B | |  | 111.88 | 71.14 | **1.57** |  | 46.86 | 71.14 | **-1.52** |  | 94.52 | 71.14 | **1.33** |
| FAM134B | 54463 | family with sequence similarity 134, member B | |  | 153.99 | 95.58 | **1.61** |  | 62.13 | 95.58 | **-1.54** |  | 70.04 | 95.58 | **0.73** |
| FAM161A | 84140 | family with sequence similarity 161, member A | |  | 52.6 | 32.27 | **1.63** |  | 14.96 | 32.27 | **-2.16** |  | 22.94 | 32.27 | **0.71** |
| GATSL3 | 652968 | GATS protein-like 3 |  |  | 43.72 | 28.73 | **1.52** |  | 14.12 | 28.73 | **-2.03** |  | 16.29 | 28.73 | **0.57** |
| HDAC11 | 79885 | histone deacetylase 11 |  |  | 245.85 | 146.62 | **1.68** |  | 97.27 | 146.62 | **-1.51** |  | 166.76 | 146.62 | **1.14** |
| HLA-DRB1 | 3123 | major histocompatibility complex, class II, DR beta 1 | |  | 344.43 | 87.05 | **3.96** |  | 55.91 | 87.05 | **-1.56** |  | 108.81 | 87.05 | **1.25** |
| HLA-DRB6 | 3128 | major histocompatibility complex, class II, DR beta 6 (pseudogene) | | | 100.4 | 45.32 | **2.22** |  | 29.77 | 45.32 | **-1.52** |  | 38.86 | 45.32 | **0.86** |
| HR | 55806 | hairless homolog (mouse) |  |  | 78.3 | 48.6 | **1.61** |  | 31.54 | 48.6 | **-1.54** |  | 40.52 | 48.6 | **0.83** |
| KBTBD6 | 89890 | kelch repeat and BTB (POZ) domain containing 6 | |  | 285.77 | 168.4 | **1.70** |  | 106.29 | 168.4 | **-1.58** |  | 170.16 | 168.4 | **1.01** |
| KCNK5 | 8645 | potassium channel, subfamily K, member 5 |  |  | 203.52 | 56.13 | **3.63** |  | 21.45 | 56.13 | **-2.62** |  | 43.6 | 56.13 | **0.78** |
| MBOAT1 | 154141 | membrane bound O-acyltransferase domain containing 1 | |  | 312.55 | 191.61 | **1.63** |  | 90.4 | 191.61 | **-2.12** |  | 173.52 | 191.61 | **0.91** |
| MCM2 | 4171 | minichromosome maintenance complex component 2 | |  | 930.86 | 562.73 | **1.65** |  | 336.99 | 562.73 | **-1.67** |  | 655.51 | 562.73 | **1.16** |
| MCM5 | 4174 | minichromosome maintenance complex component 5 | |  | 91.07 | 60.35 | **1.51** |  | 36.83 | 60.35 | **-1.64** |  | 74.97 | 60.35 | **1.24** |
| MICB | 4277 | MHC class I polypeptide-related sequence B | |  | 261.11 | 154.59 | **1.69** |  | 69.66 | 154.59 | **-2.22** |  | 94.64 | 154.59 | **0.61** |
| MLF1IP | 79682 | MLF1 interacting protein |  |  | 72.5 | 48.19 | **1.50** |  | 20.92 | 48.19 | **-2.30** |  | 44.23 | 48.19 | **0.92** |
| MPPED2 | 744 | metallophosphoesterase domain containing 2 | |  | 201.19 | 45.65 | **4.41** |  | 29.86 | 45.65 | **-1.53** |  | 130.29 | 45.65 | **2.85** |
| MSMB | 4477 | microseminoprotein, beta- |  |  | 821.69 | 318.18 | **2.58** |  | 191.12 | 318.18 | **-1.66** |  | 326.83 | 318.18 | **1.03** |
| NR2F2 | 7026 | nuclear receptor subfamily 2, group F, member 2 | |  | 48.13 | 29.35 | **1.64** |  | 16.4 | 29.35 | **-1.79** |  | 34.66 | 29.35 | **1.18** |
| PEX11A | 8800 | Peroxisomal biogenesis factor 11 alpha |  |  | 276.02 | 177.19 | **1.56** |  | 97.39 | 177.19 | **-1.82** |  | 184.74 | 177.19 | **1.04** |
| RBL1 | 5933 | retinoblastoma-like 1 (p107) |  |  | 115.85 | 58.59 | **1.98** |  | 36.57 | 58.59 | **-1.60** |  | 70.08 | 58.59 | **1.20** |
| REEP1 | 65055 | receptor accessory protein 1 |  |  | 131.6 | 43.73 | **3.01** |  | 17.55 | 43.73 | **-2.49** |  | 43.84 | 43.73 | **1.00** |
| RGNEF | 64283 | Rho-guanine nucleotide exchange factor |  |  | 67.63 | 32.36 | **2.09** |  | 17.21 | 32.36 | **-1.88** |  | 22.58 | 32.36 | **0.70** |
| RHOBTB3 | 22836 | Rho-related BTB domain containing 3 |  |  | 4117.77 | 2643.47 | **1.56** |  | 1611.12 | 2643.47 | **-1.64** |  | 2444.12 | 2643.47 | **0.92** |
| RLN2 | 6019 | relaxin 2 |  |  | 225.93 | 135.25 | **1.67** |  | 59.33 | 135.25 | **-2.28** |  | 68.67 | 135.25 | **0.51** |
| SLC9A3R1 | 9368 | solute carrier family 9 (sodium/hydrogen exchanger), member 3 regulator 1 | | | 3243.19 | 1096.73 | **2.96** |  | 696.31 | 1096.73 | **-1.58** |  | 1802.14 | 1096.73 | **1.64** |
| SMOC2 | 64094 | SPARC related modular calcium binding 2 |  |  | 159.75 | 98.58 | **1.62** |  | 44.24 | 98.58 | **-2.23** |  | 83.85 | 98.58 | **0.85** |
| SOX3 | 6658 | SRY (sex determining region Y)-box 3 |  |  | 77.83 | 20.85 | **3.73** |  | 13.56 | 20.85 | **-1.54** |  | 53.85 | 20.85 | **2.58** |
| SYTL4 | 94121 | synaptotagmin-like 4 |  |  | 166.52 | 72.8 | **2.29** |  | 41.69 | 72.8 | **-1.75** |  | 67.2 | 72.8 | **0.92** |
| TSKU | 25987 | tsukushin |  |  | 232.62 | 76.71 | **3.03** |  | 45.93 | 76.71 | **-1.67** |  | 247.27 | 76.71 | **3.22** |
| TTC39A | 22996 | tetratricopeptide repeat domain 39A |  |  | 595.16 | 384.6 | **1.55** |  | 254.63 | 384.6 | **-1.51** |  | 276.46 | 384.6 | **0.72** |
| WDHD1 | 11169 | WD repeat and HMG-box DNA binding protein 1 | |  | 202.03 | 123.07 | **1.64** |  | 80.9 | 123.07 | **-1.52** |  | 144.91 | 123.07 | **1.18** |
| XYLT1 | 64131 | xylosyltransferase I |  |  | 207.91 | 105.71 | **1.97** |  | 61.42 | 105.71 | **-1.72** |  | 106.44 | 105.71 | **1.01** |
|  |  |  |  |  |  |  |  |  |  |  |  |  |  |  |  |
| **E2 downregulated genes - EGF upregulated genes** | | |  |  |  |  |  |  |  |  |  |  |  |  |  |
| **Gene symbol** | **EntrezID** | **Description** |  |  | **E2** | **control** | **Fold-change** |  | **EGF** | **control** | **Fold-change** |  | **EGF + E2** | **control** | **Fold-change** |
| AKAP13 | 11214 | A kinase (PRKA) anchor protein 13 |  |  | 27.88 | 44.75 | **-1.61** |  | 84.45 | 44.75 | **1.89** |  | 59.09 | 44.75 | **1.32** |
| ARHGAP29 | 9411 | Rho GTPase activating protein 29 |  |  | 134.69 | 238.77 | **-1.77** |  | 443.85 | 238.77 | **1.86** |  | 231.43 | 238.77 | **0.97** |
| ARID5B | 84159 | AT rich interactive domain 5B (MRF1-like) |  |  | 127.13 | 253.02 | **-1.99** |  | 515.45 | 253.02 | **2.04** |  | 327 | 253.02 | **1.29** |
| ATP1B1 | 481 | ATPase, Na+/K+ transporting, beta 1 polypeptide | |  | 550.78 | 882.92 | **-1.60** |  | 2194.24 | 882.92 | **2.49** |  | 1343.57 | 882.92 | **1.52** |
| ATP2B1 | 490 | ATPase, Ca++ transporting, plasma membrane 1 | |  | 1091.3 | 1842.25 | **-1.69** |  | 3170.17 | 1842.25 | **1.72** |  | 2392.81 | 1842.25 | **1.30** |
| ATXN1 | 6310 | ataxin 1 |  |  | 43.33 | 89.55 | **-2.07** |  | 490.38 | 89.55 | **5.48** |  | 411 | 89.55 | **4.59** |
| AXIN2 | 8313 | axin 2 |  |  | 11.84 | 18.9 | **-1.60** |  | 35.5 | 18.9 | **1.88** |  | 20.22 | 18.9 | **1.07** |
| BHLHE40 | 8553 | basic helix-loop-helix family, member e40 |  |  | 509.47 | 810.45 | **-1.59** |  | 1364.71 | 810.45 | **1.68** |  | 1221.86 | 810.45 | **1.51** |
| BMP7 | 655 | bone morphogenetic protein 7 |  |  | 65.01 | 174.44 | **-2.68** |  | 346.82 | 174.44 | **1.99** |  | 149.52 | 174.44 | **0.86** |
| BMPR1B | 658 | bone morphogenetic protein receptor, type IB | |  | 35.75 | 70.69 | **-1.98** |  | 119.02 | 70.69 | **1.68** |  | 82.45 | 70.69 | **1.17** |
| CACHD1 | 57685 | cache domain containing 1 |  |  | 20.09 | 35.02 | **-1.74** |  | 71.81 | 35.02 | **2.05** |  | 42.88 | 35.02 | **1.22** |
| CASK | 8573 | calcium/calmodulin-dependent serine protein kinase (MAGUK family) | | | 23.8 | 37.51 | **-1.58** |  | 88.94 | 37.51 | **2.37** |  | 54.36 | 37.51 | **1.45** |
| CBLB | 868 | Cas-Br-M (murine) ecotropic retroviral transforming sequence b | | | 19.25 | 30.08 | **-1.56** |  | 65.69 | 30.08 | **2.18** |  | 51.38 | 30.08 | **1.71** |
| CCDC83 | 220047 | coiled-coil domain containing 83 |  |  | 31.29 | 81.29 | **-2.60** |  | 456.68 | 81.29 | **5.62** |  | 156.59 | 81.29 | **1.93** |
| CDYL2 | 124359 | chromodomain protein, Y-like 2 |  |  | 32.45 | 52.99 | **-1.63** |  | 85.15 | 52.99 | **1.61** |  | 61.38 | 52.99 | **1.16** |
| CLDN1 | 9076 | claudin 1 |  |  | 29.48 | 57.36 | **-1.95** |  | 561.6 | 57.36 | **9.79** |  | 232.2 | 57.36 | **4.05** |
| CRIM1 | 51232 | cysteine rich transmembrane BMP regulator 1 (chordin-like) | |  | 76.96 | 133.75 | **-1.74** |  | 252.95 | 133.75 | **1.89** |  | 188.21 | 133.75 | **1.41** |
| CSGALNACT1 | 55790 | chondroitin sulfate N-acetylgalactosaminyltransferase 1 | |  | 14.31 | 39.62 | **-2.77** |  | 194.44 | 39.62 | **4.91** |  | 94.56 | 39.62 | **2.39** |
| CXCR4 | 7852 | chemokine (C-X-C motif) receptor 4 |  |  | 17.27 | 28.95 | **-1.68** |  | 128.84 | 28.95 | **4.45** |  | 40.69 | 28.95 | **1.41** |
| DSC2 | 1824 | desmocollin 2 |  |  | 91.52 | 144.54 | **-1.58** |  | 289.54 | 144.54 | **2.00** |  | 188.89 | 144.54 | **1.31** |
| EFR3B | 22979 | EFR3 homolog B (S. cerevisiae) |  |  | 136.71 | 206.33 | **-1.51** |  | 319.76 | 206.33 | **1.55** |  | 190.1 | 206.33 | **0.92** |
| EGLN3 | 112399 | egl nine homolog 3 (C. elegans) |  |  | 31.59 | 63.4 | **-2.01** |  | 233.92 | 63.4 | **3.69** |  | 85.53 | 63.4 | **1.35** |
| ELL2 | 22936 | elongation factor, RNA polymerase II, 2 |  |  | 48.84 | 80.67 | **-1.65** |  | 284.88 | 80.67 | **3.53** |  | 221.6 | 80.67 | **2.75** |
| ENPP1 | 5167 | ectonucleotide pyrophosphatase/phosphodiesterase 1 | |  | 28.13 | 55.31 | **-1.97** |  | 103.38 | 55.31 | **1.87** |  | 63.25 | 55.31 | **1.14** |
| EPAS1 | 2034 | endothelial PAS domain protein 1 |  |  | 65.64 | 174.54 | **-2.66** |  | 498.59 | 174.54 | **2.86** |  | 252.9 | 174.54 | **1.45** |
| EPHA4 | 2043 | EPH receptor A4 |  |  | 12.44 | 26.81 | **-2.16** |  | 53.09 | 26.81 | **1.98** |  | 38.24 | 26.81 | **1.43** |
| F2RL1 | 2150 | coagulation factor II (thrombin) receptor-like 1 | |  | 31.26 | 62.97 | **-2.01** |  | 656.46 | 62.97 | **10.42** |  | 253.54 | 62.97 | **4.03** |
| FAM100B | 283991 | family with sequence similarity 100, member B | |  | 88.78 | 156.23 | **-1.76** |  | 245.46 | 156.23 | **1.57** |  | 193.91 | 156.23 | **1.24** |
| FBXO32 | 114907 | F-box protein 32 |  |  | 17.3 | 34.06 | **-1.97** |  | 64.64 | 34.06 | **1.90** |  | 51.13 | 34.06 | **1.50** |
| GALNT10 | 55568 | UDP-N-acetyl-alpha-D-galactosamine:polypeptide N-acetylgalactosaminyltransferase 10 (GalNAc-T10) | | | 21.99 | 39.76 | **-1.81** |  | 71.19 | 39.76 | **1.79** |  | 30.21 | 39.76 | **0.76** |
| GPRC5A | 9052 | G protein-coupled receptor, family C, group 5, member A | |  | 107.3 | 244.32 | **-2.28** |  | 622.04 | 244.32 | **2.55** |  | 378.04 | 244.32 | **1.55** |
| GULP1 | 51454 | GULP, engulfment adaptor PTB domain containing 1 | |  | 25.93 | 41.72 | **-1.61** |  | 79.8 | 41.72 | **1.91** |  | 47.89 | 41.72 | **1.15** |
| HDAC5 | 10014 | histone deacetylase 5 |  |  | 73.62 | 136.57 | **-1.86** |  | 308.58 | 136.57 | **2.26** |  | 154.38 | 136.57 | **1.13** |
| ID2 | 3398 | inhibitor of DNA binding 2, dominant negative helix-loop-helix protein | | | 16.55 | 27.84 | **-1.68** |  | 47.11 | 27.84 | **1.69** |  | 16.06 | 27.84 | **0.58** |
| INPP4B | 8821 | inositol polyphosphate-4-phosphatase, type II, 105kDa | |  | 429.13 | 716.47 | **-1.67** |  | 1343.84 | 716.47 | **1.88** |  | 1049.13 | 716.47 | **1.46** |
| ITGB6 | 3694 | integrin, beta 6 |  |  | 16.87 | 37.66 | **-2.23** |  | 227.85 | 37.66 | **6.05** |  | 97.54 | 37.66 | **2.59** |
| ITPR1 | 3708 | inositol 1,4,5-triphosphate receptor, type 1 |  |  | 87.12 | 174.99 | **-2.01** |  | 272.01 | 174.99 | **1.55** |  | 166.89 | 174.99 | **0.95** |
| KIAA1199 | 57214 | KIAA1199 |  |  | 46.07 | 69.33 | **-1.50** |  | 601.76 | 69.33 | **8.68** |  | 418.27 | 69.33 | **6.03** |
| KLF6 | 1316 | Kruppel-like factor 6 |  |  | 125.34 | 188.1 | **-1.50** |  | 339.49 | 188.1 | **1.80** |  | 235.25 | 188.1 | **1.25** |
| KRT20 | 54474 | keratin 20 |  |  | 7.36 | 13 | **-1.77** |  | 92.56 | 13 | **7.12** |  | 23.22 | 13 | **1.79** |
| LGALS8 | 3964 | lectin, galactoside-binding, soluble, 8 |  |  | 87.2 | 134.8 | **-1.55** |  | 210.69 | 134.8 | **1.56** |  | 165.93 | 134.8 | **1.23** |
| LIPH | 200879 | lipase, member H |  |  | 15.77 | 41.55 | **-2.63** |  | 65.99 | 41.55 | **1.59** |  | 30.08 | 41.55 | **0.72** |
| LMO7 | 4008 | LIM domain 7 |  |  | 22.24 | 53.73 | **-2.42** |  | 401.96 | 53.73 | **7.48** |  | 169.99 | 53.73 | **3.16** |
| LOC645431 | 645431 | hypothetical LOC645431 |  |  | 198.98 | 380.23 | **-1.91** |  | 741.56 | 380.23 | **1.95** |  | 511.83 | 380.23 | **1.35** |
| LXN | 56925 | latexin |  |  | 1917.98 | 3740.83 | **-1.95** |  | 6085.03 | 3740.83 | **1.63** |  | 3636.46 | 3740.83 | **0.97** |
| MIR21 | 406991 | microRNA 21 |  |  | 202.18 | 411.57 | **-2.04** |  | 1338.7 | 411.57 | **3.25** |  | 753.4 | 411.57 | **1.83** |
| MYADM | 91663 | myeloid-associated differentiation marker |  |  | 127.32 | 234.34 | **-1.84** |  | 440.6 | 234.34 | **1.88** |  | 210.81 | 234.34 | **0.90** |
| NFATC2 | 4773 | Nuclear factor of activated T-cells, cytoplasmic, calcineurin-dependent 2 | | | 90.58 | 136.71 | **-1.51** |  | 220.02 | 136.71 | **1.61** |  | 102.12 | 136.71 | **0.75** |
| NFKBIZ | 64332 | nuclear factor of kappa light polypeptide gene enhancer in B-cells inhibitor, zeta | | | 79.88 | 140.61 | **-1.76** |  | 290.3 | 140.61 | **2.06** |  | 118.45 | 140.61 | **0.84** |
| NHS | 4810 | Nance-Horan syndrome (congenital cataracts and dental anomalies) | | | 29.55 | 44.61 | **-1.51** |  | 293.4 | 44.61 | **6.58** |  | 151.09 | 44.61 | **3.39** |
| NME7 | 29922 | non-metastatic cells 7, protein expressed in (nucleoside-diphosphate kinase) | | | 55.14 | 86.56 | **-1.57** |  | 245.03 | 86.56 | **2.83** |  | 122.76 | 86.56 | **1.42** |
| NT5E | 4907 | 5'-nucleotidase, ecto (CD73) |  |  | 17.93 | 31.99 | **-1.78** |  | 59.47 | 31.99 | **1.86** |  | 24.56 | 31.99 | **0.77** |
| NVL | 4931 | nuclear VCP-like |  |  | 137.06 | 213.77 | **-1.56** |  | 565.39 | 213.77 | **2.64** |  | 331.68 | 213.77 | **1.55** |
| OSBPL6 | 114880 | oxysterol binding protein-like 6 |  |  | 12.79 | 19.89 | **-1.56** |  | 37.24 | 19.89 | **1.87** |  | 32.99 | 19.89 | **1.66** |
| OXTR | 5021 | oxytocin receptor |  |  | 20.32 | 37.93 | **-1.87** |  | 125.03 | 37.93 | **3.30** |  | 36.05 | 37.93 | **0.95** |
| PAPSS2 | 9060 | 3'-phosphoadenosine 5'-phosphosulfate synthase 2 | |  | 16.96 | 25.54 | **-1.51** |  | 60.37 | 25.54 | **2.36** |  | 56.71 | 25.54 | **2.22** |
| PAWR | 5074 | PRKC, apoptosis, WT1, regulator |  |  | 522.76 | 803.89 | **-1.54** |  | 1222.96 | 803.89 | **1.52** |  | 936.95 | 803.89 | **1.17** |
| PGM2L1 | 283209 | phosphoglucomutase 2-like 1 |  |  | 14.67 | 22.87 | **-1.56** |  | 61.41 | 22.87 | **2.69** |  | 39.78 | 22.87 | **1.74** |
| PLEKHH2 | 130271 | pleckstrin homology domain containing, family H (with MyTH4 domain) member 2 | | | 15.8 | 30.89 | **-1.96** |  | 84.18 | 30.89 | **2.73** |  | 31.37 | 30.89 | **1.02** |
| PLK2 | 10769 | polo-like kinase 2 (Drosophila) |  |  | 556.57 | 968.29 | **-1.74** |  | 1467.25 | 968.29 | **1.52** |  | 680.96 | 968.29 | **0.70** |
| PMEPA1 | 56937 | prostate transmembrane protein, androgen induced 1 | |  | 401.99 | 633.63 | **-1.58** |  | 1078.39 | 633.63 | **1.70** |  | 661.79 | 633.63 | **1.04** |
| PXN | 5829 | paxillin |  |  | 58.3 | 89.75 | **-1.54** |  | 179.55 | 89.75 | **2.00** |  | 136.52 | 89.75 | **1.52** |
| RASD1 | 51655 | RAS, dexamethasone-induced 1 |  |  | 75.88 | 212.16 | **-2.80** |  | 517.04 | 212.16 | **2.44** |  | 251.42 | 212.16 | **1.19** |
| RBMS1 | 5937 | RNA binding motif, single stranded interacting protein 1 | |  | 286.57 | 435.06 | **-1.52** |  | 873.74 | 435.06 | **2.01** |  | 634.92 | 435.06 | **1.46** |
| RND1 | 27289 | Rho family GTPase 1 |  |  | 64.5 | 176.43 | **-2.74** |  | 294.94 | 176.43 | **1.67** |  | 126.57 | 176.43 | **0.72** |
| RND3 | 390 | Rho family GTPase 3 |  |  | 45.49 | 74.78 | **-1.64** |  | 176.72 | 74.78 | **2.36** |  | 104.86 | 74.78 | **1.40** |
| SEMA4B | 10509 | sema domain, immunoglobulin domain (Ig), transmembrane domain (TM) and short cytoplasmic domain, (semaphorin) 4B | | | 135.36 | 217.52 | **-1.61** |  | 356.87 | 217.52 | **1.64** |  | 230.45 | 217.52 | **1.06** |
| SFMBT2 | 57713 | Scm-like with four mbt domains 2 |  |  | 25.43 | 51.44 | **-2.02** |  | 137.9 | 51.44 | **2.68** |  | 72.28 | 51.44 | **1.41** |
| SMAD3 | 4088 | SMAD family member 3 |  |  | 240.86 | 539.28 | **-2.24** |  | 1216.63 | 539.28 | **2.26** |  | 664.12 | 539.28 | **1.23** |
| STARD4 | 134429 | StAR-related lipid transfer (START) domain containing 4 | |  | 24.85 | 37.35 | **-1.50** |  | 70.95 | 37.35 | **1.90** |  | 48.56 | 37.35 | **1.30** |
| SYTL2 | 54843 | synaptotagmin-like 2 |  |  | 951.84 | 2140.18 | **-2.25** |  | 4674.25 | 2140.18 | **2.18** |  | 3152.98 | 2140.18 | **1.47** |
| TBC1D8 | 11138 | TBC1 domain family, member 8 (with GRAM domain) | |  | 94.06 | 154.29 | **-1.64** |  | 255.45 | 154.29 | **1.66** |  | 150.94 | 154.29 | **0.98** |
| TFPI | 7035 | tissue factor pathway inhibitor (lipoprotein-associated coagulation inhibitor) | | | 606.39 | 1492.14 | **-2.46** |  | 2375.76 | 1492.14 | **1.59** |  | 1260.07 | 1492.14 | **0.84** |
| TGFB2 | 7042 | transforming growth factor, beta 2 |  |  | 13.03 | 22.85 | **-1.75** |  | 36.61 | 22.85 | **1.60** |  | 16.82 | 22.85 | **0.74** |
| TM4SF1 | 4071 | transmembrane 4 L six family member 1 |  |  | 16.95 | 39.65 | **-2.34** |  | 2228.25 | 39.65 | **56.20** |  | 942.22 | 39.65 | **23.76** |
| TMEM45B | 120224 | transmembrane protein 45B |  |  | 51.08 | 119.38 | **-2.34** |  | 417.1 | 119.38 | **3.49** |  | 172.6 | 119.38 | **1.45** |
| TMEM49 | 81671 | Transmembrane protein 49 |  |  | 16.15 | 27.08 | **-1.68** |  | 112.47 | 27.08 | **4.15** |  | 66.48 | 27.08 | **2.45** |
| TNFRSF11B | 4982 | tumor necrosis factor receptor superfamily, member 11b | |  | 19.94 | 68.91 | **-3.46** |  | 466.17 | 68.91 | **6.76** |  | 179.99 | 68.91 | **2.61** |
| WNT3 | 7473 | wingless-type MMTV integration site family, member 3 | |  | 26.38 | 41.96 | **-1.59** |  | 63.3 | 41.96 | **1.51** |  | 47.89 | 41.96 | **1.14** |
