# Supplementary material for: Epidermal growth factor receptor signalling in human breast cancer cells operates parallel to estrogen receptor α signalling and results in tamoxifen insensitive proliferation
Source: BMC Cancer. 2014 Apr 23;14:283. doi: 10.1186/1471-2407-14-283 (PMC4021213; doi:10.1186/1471-2407-14-283)
Supplement: Additional file 8: Table S3 — E2 and EGF induced changes in gene expression related to cell proliferation. [file 1471-2407-14-283-S8.doc]

**Additional Table S3.** *E2 and EGF induced changes in gene expression related to cell proliferation*

| **RELATED TO E2 INDUCED PROLIFERATION** | |  | | --- | |  | **E2** |  |  | |  | | --- | | **EGF** |  |  | |  | | --- | | **E2+EGF** |  |
| --- | --- | --- | --- | --- | --- | --- | --- | --- | --- | --- | --- | --- | --- | --- | --- |
| ***Gene symbol*** |  | ***Fold change E2*** | ***Fold change E2+TAM*** | ***% inhibition TAM*** |  | ***Fold change EGF*** | ***Fold change EGF+TAM*** | ***% inhibition TAM*** |  | ***Fold change EGF+E2*** | ***Fold change E2+EGF+TAM*** | ***% inhibition TAM*** |
| **positive regulators for E2F activation** | | |  |  |  |  |  |  |  |  |  |  |
| MYC |  | 1.63 | 1.01 | 98.4 |  | 1.02 | 1.30 |  |  | 1.90 | 1.48 | 46.9 |
| E2F3 |  | 1.43 | 1.07 | 84.1 |  | 1.39 | 1.32 | 18.3 |  | 1.54 | 1.45 | 17.9 |
| E2F5 |  | 1.31 | 1.09 | 69.4 |  | 1.49 | 1.47 | 3.6 |  | 1.66 | 1.50 | 24.1 |
| CDC25A |  | 1.71 | 1.13 | 81.8 |  | -1.28 | -1.46 |  |  | 1.47 | 1.07 | 84.9 |
| **anti-proliferative** | |  |  |  |  |  |  |  |  |  |  |  |
| CDKN1A |  | -1.78 | -1.59 | 15.5 |  | 1.25 | 1.03 | 89.9 |  | -1.29 | -1.10 | 56.9 |
| BTG2 |  | -2.95 | -1.57 | 45.2 |  | -1.22 | -1.13 | 34.9 |  | -2.17 | -1.50 | 40.5 |
| SUV420H1 |  | -1.47 | -1.14 | 61.8 |  | -1.25 | -1.27 |  |  | -1.68 | -1.40 | 34.9 |
| SMAD3 |  | -2.24 | -1.88 | 15.2 |  | 2.26 | 2.06 | 15.3 |  | 1.23 | 1.65 |  |
| CDKN2B |  | -3.83 | -2.21 | 26.0 |  | -1.17 | -1.20 |  |  | -2.81 | -2.2 | 16.0 |
| TGFB1 |  | -1.26 | -1.11 | 50.6 |  | 1.45 | 1.45 | 0.6 |  | 1.09 | 1.24 |  |
| TGFBI |  | -1.18 | -1.02 | 85.0 |  | 2.53 | 2.04 | 32.2 |  | 1.34 | 1.47 |  |
| TGFB2 |  | -1.75 | -1.44 | 28.9 |  | 1.60 | 1.61 |  |  | -1.36 | 1.01 |  |
| TGFB3 |  | -2.39 | -2.11 | 9.6 |  | -3.05 | -3.67 |  |  | -5.43 | -5.10 | 1.3 |
| **known E2 induced genes** | | |  |  |  |  |  |  |  |  |  |  |
| CCNA1 |  | 1.19 | 1.11 | 39.9 |  | 4.99 | 4.87 | 3.1 |  | 3.83 | 4.04 |  |
| CCND1 |  | 1.86 | 1.00 | 100.3 |  | 1.07 | 1.03 | 62.6 |  | 1.94 | 1.23 | 75.4 |
| CCNE2 |  | 1.48 | 1.01 | 97.5 |  | -1,22 | -1.58 |  |  | 1.33 | -1.20 | 154.4 |
| TK1 |  | 1.24 | 1.07 | 72.4 |  | -1.63 | -1.89 |  |  | -1.04 | -1.50 |  |
| PCNA |  | 1.55 | 1.13 | 77.0 |  | -1.37 | -1.47 |  |  | 1.09 | -1.20 |  |
| DHFR |  | 1.30 | 1.04 | 88.3 |  | -1.29 | -1.33 |  |  | -1.15 | -1.40 |  |
| EZH2 |  | 1.28 | 1.05 | 80.1 |  | 1.00 | -1.03 |  |  | 1.19 | 1.04 | 78.0 |
| CDC6 |  | 2.13 | 1.18 | 84.3 |  | -1.21 | -1.63 |  |  | 1.97 | 1.20 | 79.9 |
| MCM5 |  | 1.51 | 1.14 | 72.8 |  | -1.64 | -1.73 |  |  | 1.24 | -1.20 |  |
| MCM7 |  | 1.60 | 1.10 | 83.1 |  | 1.04 | -1.16 |  |  | 1.68 | 1.08 | 88.3 |
| MCM4 |  | 1.56 | 1.14 | 75.0 |  | 1.08 | 1.03 | 67.0 |  | 1.65 | 1.21 | 68.1 |
|  |  |  |  |  |  |  |  |  |  |  |  |  |
| **growth factor ligands and receptors** | | |  |  |  |  |  |  |  |  |  |  |
| ARTN |  | 1.41 | -1.02 | 103.7 |  | 1.03 | -1.17 |  |  | 1.92 | 1.11 | 87.9 |
| TGFA |  | 2.48 | -1.06 | 103.9 |  | 1.17 | 1.08 | 55.0 |  | 2.31 | 1.23 | 82.5 |
| HBEGF |  | -1.19 | -1.05 | 72.3 |  | 3.94 | 1.89 | 69.6 |  | 1.77 | 2.18 |  |
| HBEGF |  | -1.20 | -1.08 | 56.6 |  | 2.62 | 1.88 | 46.0 |  | 1.59 | 1.81 |  |
| AREG |  | 2.29 | 1.15 | 88.2 |  | 5.85 | 5.34 | 10.6 |  | 7.44 | 7.23 | 3.3 |
| VEGFA |  | 1.41 | 1.13 | 69.3 |  | 1.60 | 1.66 |  |  | 1.87 | 1.83 | 5.1 |
| AREGB |  | 1.24 | -1.06 | 123.8 |  | 2.27 | 1.91 | 28.5 |  | 2.47 | 2.18 | 19.5 |
|  |  |  |  |  |  |  |  |  |  |  |  |  |
| **apoptosis inducing** | |  |  |  |  |  |  |  |  |  |  |  |
| SGPL1 |  | -1.26 | -1.06 | 72.9 |  | -1.34 | -1.27 | 16.4 |  | -1.51 | -1.40 | 20.3 |
| BIK |  | -2.88 | -1.50 | 48.6 |  | -1.43 | -1.36 | 12.3 |  | -2.29 | -1.70 | 26.0 |
| BMF |  | -4.67 | -2.33 | 27.5 |  | -1.98 | -1.62 | 22.4 |  | -4.25 | -3.50 | 6.5 |
| APAF1 |  | -1.15 | -1.05 | 63.4 |  | -1.97 | -1.74 | 13.8 |  | -1.90 | -1.70 | 13.1 |
| **apoptose preventing** | |  |  |  |  |  |  |  |  |  |  |  |
| FAIM3 |  | 2.51 | 1.92 | 38.8 |  | 1.74 | 1.10 | 86.7 |  | 3.74 | 2.85 | 32.3 |
| BCL2 |  | 2.03 | 1.76 | 26.1 |  | -1.96 | -1.54 | 28.1 |  | -1.12 | -1.20 |  |
| IER3 |  | 1.28 | 2.09 |  |  | 1.08 | 1.62 |  |  | -1.04 | 1.69 |  |
| HSPB8 |  | 4.82 | 3.25 | 41.2 |  | 1.56 | 1.39 | 30.2 |  | 5.42 | 4.08 | 30.2 |
| **effector caspases** | |  |  |  |  |  |  |  |  |  |  |  |
| CASP7 |  | 1.48 | 3.63 |  |  | 1.13 | 1.17 |  |  | 1.62 | 3.45 |  |
| CASP2 |  | 1.29 | 1.09 | 70.1 |  | -1.08 | -1.10 |  |  | 1.12 | 1.02 | 85.0 |
| **initiator caspases** | |  |  |  |  |  |  |  |  |  |  |  |
| CASP9 |  | -1.48 | 1.00 | 100.4 |  | -1.31 | -1.18 | 35.4 |  | -1.75 | -1.40 | 38.7 |
| CASP6 |  | -1.22 | -1.13 | 34.5 |  | -1.16 | -1.16 |  |  | -1.42 | -1.30 | 17.5 |
|  |  |  |  |  |  |  |  |  |  |  |  |  |
| **repressed putative tumor suppressors** | | |  |  |  |  |  |  |  |  |  |  |
| BLNK |  | -3.41 | -1.78 | 38.2 |  | -2.80 | -2.98 |  |  | -7.31 | -5.40 | 5.7 |
| LATS2 |  | -1.52 | -1.24 | 43.5 |  | 1.05 | 1.07 |  |  | -1.38 | -1.10 | 70.2 |
| RPRM |  | -1.70 | -1.34 | 38.2 |  | -1.17 | -1.21 |  |  | -1.68 | -1.50 | 20.6 |
|  |  |  |  |  |  |  |  |  |  |  |  |  |
| **induced proto-oncogenes** | | |  |  |  |  |  |  |  |  |  |  |
| RET |  | 9.06 | 4.20 | 60.3 |  | 1.73 | 1.55 | 24.8 |  | 8.57 | 4.66 | 51.7 |
| GFRA2 |  | 1.82 | 1.26 | 68.7 |  | 2.37 | 2.29 | 6.0 |  | 11.05 | 4.01 | 70.1 |
| MERTK |  | 2.87 | 1.14 | 92.6 |  | 1.06 | 1.07 |  |  | 4.14 | 1.05 | 98.3 |
|  |  |  |  |  |  |  |  |  |  |  |  |  |
| **Cytokine signalling** | |  |  |  |  |  |  |  |  |  |  |  |
| CXCR4 |  | -1.21 | -1.66 |  |  | 5.30 | 4.24 | 24.8 |  | 2.04 | 2.45 |  |
| CXCL12 |  | 14.53 | 4.51 | 74.1 |  | 2.24 | 1.89 | 27.8 |  | 12.77 | 5.63 | 60.7 |
| IL20 |  | 4.11 | 2.25 | 59.8 |  | 2.20 | 1.41 | 66.0 |  | 6.91 | 5.26 | 28.0 |

|  |  |  |  |  |  |  |  |  |  |  |  |  |
| --- | --- | --- | --- | --- | --- | --- | --- | --- | --- | --- | --- | --- |
| **RELATED TO EGF INDUCED PROLIFERATION** | |  | | --- | |  | **E2** |  |  |  | **EGF** |  |  | |  | | --- | | **E2 + EGF** |  |
| ***Gene symbol*** |  | ***Fold change E2*** | ***Fold change E2+TAM*** | ***% inhibition TAM*** |  | ***Fold change EGF*** | ***Fold change EGF+TAM*** | ***% inhibition TAM*** |  | ***Fold change EGF+E2*** | ***Fold change E2+EGF+TAM*** | ***% inhibition TAM*** |
| MITF |  | 1.46 | 1.32 | 31.1 |  | 2.91 | 3.11 |  |  | 2.63 | 3.23 |  |
| ELF4 |  | 1.01 | -1.11 |  |  | 1.82 | 1.83 |  |  | 1.82 | 1.75 | 8.6 |
| CREM |  | 1.03 | -1.06 |  |  | 1.70 | 1.81 |  |  | 1.50 | 1.52 |  |
| PRNP |  | 1.21 | 1.09 | 57.4 |  | 2.09 | 1.99 | 8.5 |  | 2.16 | 2.13 | 2.7 |
| SOX9 |  | 1.34 | -1.04 | 110.2 |  | 3.37 | 2.81 | 23.3 |  | 5.15 | 4.67 | 11.5 |
| MAFF |  | -1.07 | -1.38 |  |  | 6.13 | 6.40 |  |  | 3.48 | 5.30 |  |
| FOS |  | 1.45 | -1.01 | 102.8 |  | 2.13 | 1.58 | 49.0 |  | 1.65 | 2.09 |  |
| FOSB |  | -1.11 | -1.01 | 86.8 |  | 1.90 | 1.55 | 38.7 |  | 1.35 | 1.29 | 18.1 |
| FOSL1 |  | 1.21 | 1.06 | 70.1 |  | 2.96 | 2.18 | 40.1 |  | 2.37 | 1.99 | 28.0 |
| JUN |  | 1.15 | 1.16 |  |  | 1.63 | 1.55 | 12.1 |  | 1.59 | 1.76 |  |
| JUNB |  | -1.50 | -1.52 |  |  | 2.14 | 2.31 |  |  | 1.57 | 1.97 |  |
| CAPN2 |  | -1.24 | -1.12 | 43.5 |  | 2.61 | 2.81 |  |  | 2.43 | 2.65 |  |
| EGR1 |  | 1.05 | -1.22 |  |  | 2.56 | 2.48 | 5.7 |  | 2.26 | 2.65 |  |
| CLDN1 |  | -1.95 | -1.79 | 9.1 |  | 9.79 | 8.84 | 10.8 |  | 4.05 | 6.25 |  |
| SMAD3 |  | -2.24 | -1.88 | 15.2 |  | 2.26 | 2.06 | 15.3 |  | 1.23 | 1.65 |  |
| TGFBR2 |  | -1.11 | -1.07 | 35.5 |  | 4.54 | 4.73 |  |  | 3.96 | 4.63 |  |
| TGFBI |  | -1.18 | -1.02 | 85.0 |  | 2.53 | 2.04 | 32.2 |  | 1.34 | 1.47 |  |
| TGFB2 |  | -1.75 | -1.44 | 28.9 |  | 1.60 | 1.61 |  |  | -1.36 | 1.01 |  |
| TGFB1 |  | -1.26 | -1.11 | 50.6 |  | 1.45 | 1.45 | 0.6 |  | 1.09 | 1.24 |  |
| BMP6 |  | 1.06 | -1.14 |  |  | 2.12 | 2.33 |  |  | 1.88 | 2.38 |  |
| BMP7 |  | -2.68 | -2.11 | 16.0 |  | 1.99 | 2.62 |  |  | -1.17 | 1.39 |  |
| ELF4 |  | 1.01 | -1.11 |  |  | 1.82 | 1.83 |  |  | 1.82 | 1.75 | 8.6 |
| HBEGF |  | -1.19 | -1.05 | 72.3 |  | 3.94 | 1.89 | 69.6 |  | 1.77 | 2.18 |  |
| HBEGF |  | -1.20 | -1.08 | 56.6 |  | 2.62 | 1.88 | 46.0 |  | 1.59 | 1.81 |  |
|  |  |  |  |  |  |  |  |  |  |  |  |  |
| **MAPK signature** | |  |  |  |  |  |  |  |  |  |  |  |
| RELB |  | -1.11 | 1.06 |  |  | 1.61 | 1.25 | 58.8 |  | 1.31 | 1.38 |  |
| GADD45A |  | -1.20 | -1.09 | 52.0 |  | 1.84 | 1.14 | 83.7 |  | 1.10 | 1.19 |  |
| ETV5 |  | -1.04 | 1.04 |  |  | 1.60 | 1.95 |  |  | 1.95 | 2.06 |  |
| ANGPTL4 |  | -1.10 | -1.02 | 74.4 |  | 1.32 | 1.00 | 99.3 |  | -1.07 | 1.00 |  |
| TOB1 |  | 1.02 | -1.02 |  |  | -1.43 | -1.30 | 22.1 |  | -1.34 | -1.40 |  |
| PDCD4 |  | 1.21 | 1.06 | 72.7 |  | -1.61 | -1.55 | 6.0 |  | -1.40 | -1.50 |  |
| **ELK-1 targets** |  |  |  |  |  |  |  |  |  |  |  |  |
| EGR1 |  | -1.06 | -1.37 |  |  | 3.44 | 3.07 | 15.2 |  | 3.12 | 3.18 |  |
| TIMP1 |  | -1.10 | -1.07 | 22.6 |  | 1.71 | 1.69 | 3.7 |  | 1.59 | 1.67 |  |
|  |  |  |  |  |  |  |  |  |  |  |  |  |
| **SGK1** |  |  |  |  |  |  |  |  |  |  |  |  |
| SGK1 |  | 5.17 | 1.67 | 83.9 |  | 1.55 | -1.42 |  |  | 2.52 | 1.33 | 78.3 |
|  |  |  |  |  |  |  |  |  |  |  |  |  |
| **CREB targets** |  |  |  |  |  |  |  |  |  |  |  |  |
| MCL1 |  | -1.11 | -1.11 |  |  | 1.89 | 1.71 | 20.1 |  | 1.50 | 1.82 |  |
| MLF1 |  | -1.13 | -1.10 | 17.0 |  | 1.66 | 1.65 | 2.4 |  | 1.42 | 1.55 |  |
| CRKL |  | 1.06 | 1.06 |  |  | 1.37 | -1.03 |  |  | 1.42 | 1.42 | 0.2 |
| GEM |  | 1.66 | -1.04 | 106.4 |  | 11.82 | 9.49 | 21.5 |  | 6.53 | 9.23 |  |
|  |  |  |  |  |  |  |  |  |  |  |  |  |
| **Stat3 activation** | |  |  |  |  |  |  |  |  |  |  |  |
| STAT3 |  | -1.43 | -1.30 | 24.4 |  | 1.45 | 1.30 | 32.3 |  | 1.37 | 1.28 | 23.6 |
| OSMR |  | -1.06 | -1.23 |  |  | 1.72 | 1.90 |  |  | 1.63 | 1.50 | 20.6 |
| IL6R |  | 1.04 | -1.21 |  |  | 3.93 | 3.52 | 14.2 |  | 3.20 | 4.18 |  |
| IL6ST |  | 1.13 | -1.08 |  |  | 1.23 | 1.11 | 50.7 |  | 1.19 | 1.23 |  |
| LIF |  | -1.21 | -1.19 | 8.3 |  | 5.01 | 5.02 |  |  | 2.87 | 2.64 | 12.6 |
